# Dosing time optimization of antihypertensive medications by including the circadian rhythm in pharmacokinetic-pharmacodynamic models

**DOI:** 10.1371/journal.pcbi.1010711

**Published:** 2022-11-14

**Authors:** Javiera Cortés-Ríos, Ramón C. Hermida, Maria Rodriguez-Fernandez

**Affiliations:** 1 Institute for Biological and Medical Engineering, Schools of Engineering, Medicine and Biological Sciences, Pontificia Universidad Católica de Chile, Chile; 2 Bioengineering & Chronobiology Laboratories, Atlantic Research Center for Telecommunication Technologies (atlanTTic), Universidade de Vigo, Vigo, Spain; 3 Bioengineering & Chronobiology Research Group. Galicia Sur Health Research Institute (IIS Galicia Sur). SERGAS-UVIGO, Vigo, Spain; University at Buffalo - The State University of New York, UNITED STATES

## Abstract

Blood pressure (BP) follows a circadian variation, increasing during active hours, showing a small postprandial valley and a deeper decrease during sleep. Nighttime reduction of 10–20% relative to daytime BP is defined as a dipper pattern, and a reduction of less than 10%, as a non-dipper pattern. Despite this BP variability, hypertension’s diagnostic criteria and therapeutic objectives are usually based on BP average values. Indeed, studies have shown that chrono-pharmacological optimization significantly reduces long-term cardiovascular risk if a BP dipper pattern is maintained. Changes in the effect of antihypertensive medications can be explained by circadian variations in their pharmacokinetics (PK) and pharmacodynamics (PD). Nevertheless, BP circadian variation has been scarcely included in PK-PD models of antihypertensive medications to date. In this work, we developed PK-PD models that include circadian rhythm to find the optimal dosing time (*Ta*) of first-line antihypertensive medications for dipper and non-dipper patterns. The parameters of the PK-PD models were estimated using global optimization, and models were selected according to the lowest corrected Akaike information criterion value. Simultaneously, sensitivity and identifiability analysis were performed to determine the relevance of the parameters and establish those that can be estimated. Subsequently, *Ta* parameters were optimized to maximize the effect on BP average, BP peaks, and sleep-time dip. As a result, all selected models included at least one circadian PK component, and circadian parameters had the highest sensitivity. Furthermore, *Ta* with which BP>130/80 mmHg and a dip of 10–20% are achieved were proposed when possible. We show that the optimal *Ta* depends on the therapeutic objective, the medication, and the BP profile. Therefore, our results suggest making chrono-pharmacological recommendations in a personalized way.

## Author summary

Blood pressure (BP) exhibits a circadian rhythm, with a rise during active hours, a small postprandial valley, and a deeper drop during sleep. A low nocturnal decrease in BP is a relevant cardiovascular risk factor, as are the average and peaks of BP in hypertensive patients. Studies have shown that antihypertensive medications’ effect varies at different dosing times (*Ta*). Indeed, hypertension chronotherapy significantly affects long-term cardiovascular risk. Using mathematical modeling, we established that incorporating the circadian rhythm on pharmacokinetic constants allows better prediction of the effect of antihypertensive medicines on BP. In addition, we revealed that the optimal *Ta* for antihypertensive medications is different when optimizing the nocturnal BP decrease, the average BP reduction, and the average BP peaks reduction, i.e., they are competitive optimization objectives. Moreover, models allowed us to find optimal *Ta* parameters that achieve the current therapeutic objective (BP<130/80mmHg), and, at the same time, optimal percentages of nocturnal BP decrease for different BP profiles for most antihypertensive medications studied, supporting personalized medicine for hypertension.

This is a *PLOS Computational Biology* Methods paper.

## Introduction

Arterial hypertension (AH) is a chronic medical condition characterized by a persistent increase in blood pressure (BP), and it is the modifiable risk factor that most affects mortality in the world [1]. AH can be classified according to its etiology as primary (or essential), which has an idiopathic origin, or secondary, associated with an identifiable cause. Regardless of the origin of AH, the regulatory systems involved in this condition are the renin-angiotensin-aldosterone system (RAAS), sympathetic nervous system, immune system, endothelium, and natriuretic peptides [1]. All the systems mentioned above are affected by circadian variation components, such as plasma norepinephrine, the presence of Na+ transporters in nephrons, renin activity, and the concentration of atrial natriuretic peptide angiotensin and plasma aldosterone, among others [2,3]. Consequently, the physiological mechanisms of regulation and external variables such as physical activity and feeding routine result in a circadian variation of BP [4]. The circadian variation profile of BP is characterized by a morning increase, a small postprandial valley, and a more profound decrease during night rest. A nocturnal reduction of 10–20% compared to daytime BP is defined as a dipper pattern, while a reduction of less than 10% is defined as a non-dipper pattern. The non-dipper pattern is associated with a higher cardiovascular risk than the normal fall pattern (dipper pattern) [5]. Indeed, the average BP during sleep is the most sensitive predictor of morbidity and mortality [6].

Generally, clinical guidelines recommend ambulatory BP monitoring using calibrated and approved instruments for diagnosing and monitoring AH. Then, diagnostic criteria, treatment, and therapeutic objectives are based on BP averages [7]. Although clinical guidelines indicate diagnostic criteria for AH that consider BP averages during the day and night, BP circadian variation has not been considered a therapeutic objective. Hypertension therapy typically includes lifestyle changes, such as weight loss, reduced salt intake, increased physical activity, change of diet, and reduced alcohol consumption. Often these changes are not enough to achieve the therapeutic goal, so antihypertensive medications are incorporated into the treatment. The World Health Organization recommends thiazide diuretics, angiotensin-converting enzyme (ACE) inhibitors, angiotensin II receptor blockers (ARBs), and calcium channel blockers (CCBs) as first-line antihypertensive agents [8]. Some of these are pharmacologically active drugs (non-prodrugs), and others require a metabolic step to carry out their main pharmacological effect (prodrugs), which may be circadian-phase dependent. Moreover, pharmacological targets are circadian phase-dependent; therefore, the effects of antihypertensive medications may display a circadian time dependency [9].

Daily effect variations of antihypertensive medications can be explained due to pharmacokinetics (PK) and pharmacodynamics (PD) changes. On the one hand, the variation of the different processes involved in the absorption, distribution, metabolism, and excretion (ADME) of antihypertensive medications, generates changes in PK parameters that finally produce differences in efficacy [10,11]. On the other hand, the variation of the therapeutic target during the day, for example, the expression of an enzyme or receptor, produces a variation in response to pharmacological treatment and consequently changes the dose-response curves [12]. Classical PK-PD models have been developed for β-blockers (BB), ARBs, CCBs, and ACE inhibitors [13–16], and some of the PK-PD models of antihypertensive medications have included the circadian variation of BP by coupling an indirect effect model with circadian variation [15,17,18]. But the simultaneous estimation of the circadian BP and PD parameters can lead to biased results due to the high correlation between some of them. Therefore, some BP profile variations after treatment could be explained by the effect of antihypertensive medication rather than by the intrinsic circadian variation of BP. Thus, in PD models that include BP circadian rhythm, a baseline BP function must be established prior to estimating PD parameters, as was early described by Hempel et al. [18]. Moreover, previous works indicate that not only the pharmacodynamics is altered by mechanisms of circadian variation but also the pharmacokinetic processes, mainly those administered orally [19]. However, circadian variation has not been included in PK models of antihypertensive medications. Although classic PK models do not include the circadian effects, Véronneau-Veilleux et al. [20] proposed modeling circadian fluctuations of pharmacokinetic parameters, representing each of the typical constants of a two-compartment model by a periodic function. Other authors have included periodic functions in PK models of some medications such as 5-fluorouracil, propofol, and levofloxacin [21–23], and have even modeled the variability of pharmacokinetic parameters throughout the day to optimize circadian drug infusion schedules in cancer chronotherapy [24].

Besides understanding the effect variations, according to the relevance of maintaining a normal BP profile, the interest in the chronopharmacology of hypertension has increased. The administration of antihypertensive medications is commonly recommended upon awakening to reduce daytime BP surges. However, several studies have been conducted to evaluate the effect of administering antihypertensive medications at different times of the day. Recent reviews revealed that evening dosing of ACE inhibitors benazepril, captopril, enalapril, imidapril, lisinopril, perindopril, quinapril, ramipril, spirapril, trandolapril, zofenopril; ARBs irbesartan, candesartan, olmesartan, telmisartan, valsartan; CCBs cilnidipine, isradipine, nifedipine, nisoldipine, verapamil; diuretic torasemide; α-blocker doxazosine; BBs carvedilol and nebivolol among others significantly reduce BP during night rest [9,19]. Better treatment results, adverse effects and/or increased dipping were also shown for evening than for awakening dosing of combinations; captopril/hydrochlorothiazide, enalapril/hydrochlorothiazide, trandolapril/verapamil valsartan/amlodipine, olmesartan/amlodipine, fosinopril/amlodipine, valsartan/hydrochlorothiazide, amlodipine/hydrochlorothiazide, amiloride/hydrochlorothiazide, telmisartan/amlodipine, losartan/indapamide, perindopril/indapamide, azilsartan/indapamide and valsartan/indapamide [25]. Furthermore, Hermida et al. [26] performed a prospective endpoint trial with 19,084 hypertensive patients aged 60.5 ± 13.7 and demonstrated that bedtime hypertension treatment significantly reduces cardiovascular risk. Moreover, having a non-dipper pattern is not the only risk factor associated with daily BP variability. High BP peaks, an exaggerated morning BP surge, and an elevated average BP have also been considered cardiovascular risk factors [27,28]. In addition, establishing a differentiated dosing time for extreme dippers, non-dippers, and dipper subjects have been sought to improve therapeutic outcomes in a personalized way [29–31]. Therefore, dosing time optimization of antihypertensive medications should be carried out as a multi-objective optimization problem for different starting BP profiles. Nevertheless, dosing time has not been optimized using mathematical models so far.

In this work, we propose the inclusion of circadian rhythm in PK-PD models of first-line antihypertensive medications, thereby optimizing their dosing time. We evaluated the relationship between the dosing time and dipper percentage, the effect on BP average, and the impact on BP peaks (and increased morning surge) using starting profiles of dipper and non-dipper subjects for each antihypertensive medication. Thus, we established that the optimal administration time depends on the therapeutic objective, the initial BP profile, and the antihypertensive medication. In addition, we propose administration times for each type of medication and profile (dipper or non-dipper) that allow the actual therapeutic goal BP <130/80 mmHg [8] and, at the same time, a dipper BP profile to be maintained. Therefore, we encourage personalized chrono-pharmacological recommendations for each patient.

## Results

### PK-PD models

**Fig 1:**
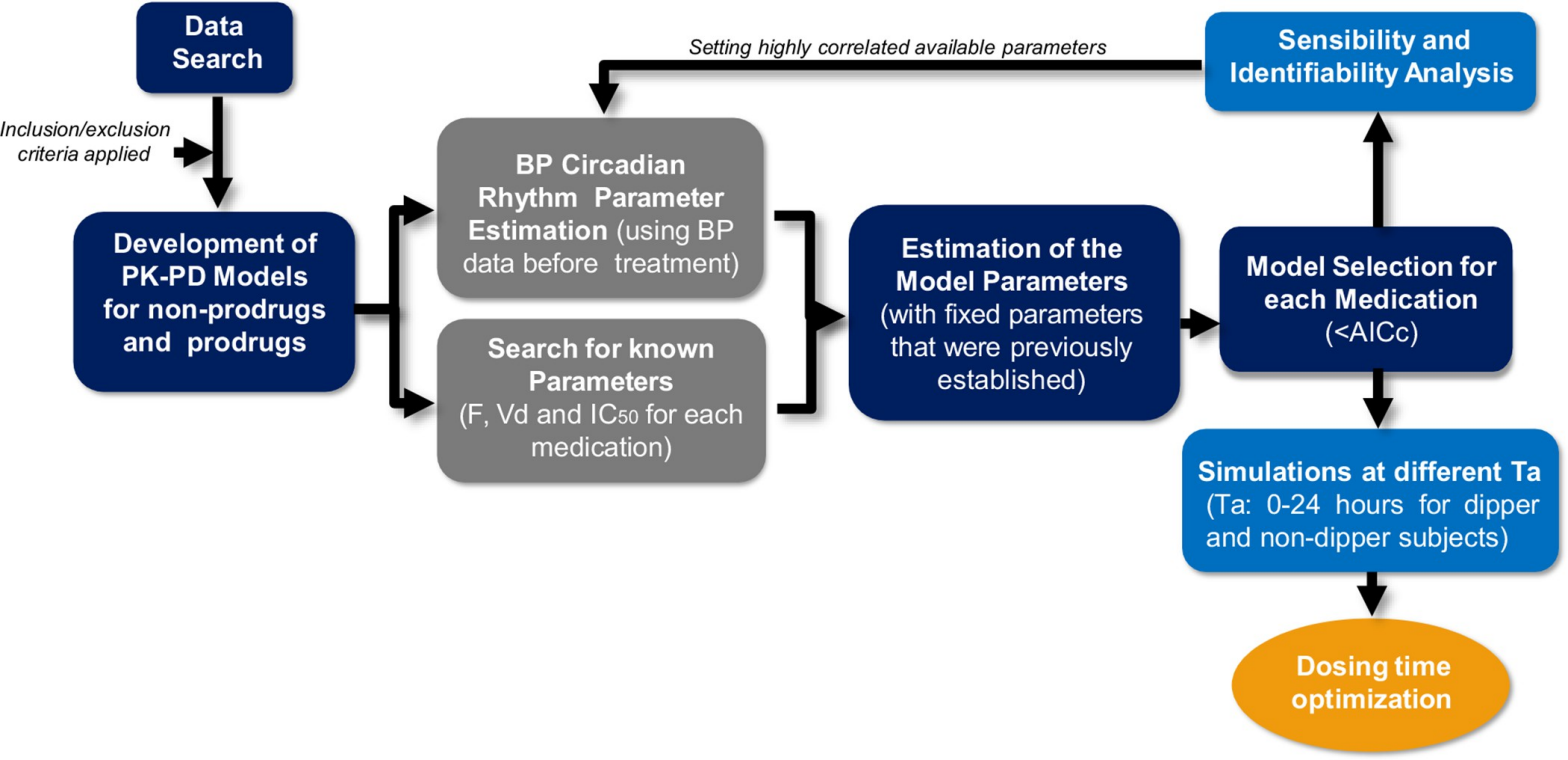
Summary scheme of the methodology employed. Inclusion and exclusion criteria and PK-PD models developed are described in the methodology section (data search and selection and PK-PD modeling sections, respectively). Preliminarily, local sensitivity and identifiability analyses of the developed PK-PD models indicated the presence of highly correlated parameters, so the circadian rhythm parameters of BP were estimated, and available pharmacokinetic parameters were also established; *F* (bioavailability), *Vd* (volume of distribution) and *IC*_*50*_ (50% inhibitory concentration). Thus, the remaining parameters of each model were estimated later. Models that include and do not include circadian kinetic constants and Hill’s coefficient in the effect equation were tested, and the model with the lowest corrected Akaike information criterion (*AICc*) was selected. Subsequently, the selected model parameters were subjected to an analysis of local sensitivity and identifiability in order to understand their relevance. Finally, simulations at different administration times (*Ta*) allowed establishing relationships between different optimization objectives and proposing optimal administration times for dipper and non-dipper subjects.

A summary of the methodology used is depicted in Fig 1. First, BP and plasma concentration data of first-line antihypertensive medications for at least two different dosing times were used to fit the models (see details of data used in Materials and Methods section, subsection Data search and selection). After finding the available data, the PK-PD models were developed (Eqs 4–13), and a preliminary sensitivity and identifiability analysis indicated the presence of highly correlated parameters. Therefore, circadian BP parameters were estimated and set before fitting the PK-PD models (Tables A and B in S1 Text), and literature PK-PD parameters were established (Table C in S1 Text). The parameters that describe the circadian rhythm of BP (parameters of Eqs 1–3 of the methodology section) were obtained using before-treatment BP data for each medication in order to set the BP circadian variation baseline [18]. Subsequently, the remaining parameters of each PK-PD model were estimated using literature data after treatment, and the model with the lowest corrected Akaike information criterion (*AICc*) among the different options (Hill coefficient *n* or not; incorporating none, only one or two kinetic constants as periodic functions) was selected. The *AICc* and objective function values (*F*_*obj*_) for all non-prodrug and prodrug models tested are reported in Tables 1 and 2, respectively. *F*_*obj*_ and *AICc* values of selected models are highlighted in bold.

**Table 1:** Objective function (*Fobj*) and corrected Akaike information criterion (*AICc*) values for non-prodrug tested models.

		Amlodipino		Nifedipino		Valsartan		Olmesartan		Telmisartan		Lisinopril	
	Models	*Fobj*	*AICc*	*Fobj*	*AICc*	*Fobj*	*AICc*	*Fobj*	*AICc*	*Fobj*	*AICc*	*Fobj*	*AICc*
No Hill coefficient (n = 1)	*No circadian Kx*	4.08	571.7	4.20	399.1	13.07	871.9	8.48	657.5	9.26	639.3	9.27	1161.1
	*Circadian Ka*	3.17	532.8	3.84	392.2	6.90	580.1	7.09	595.3	8.29	597.4	8.18	1086.4
	*Circadian Ke*	2.99	524.1	3.85	392.6	6.61	566.4	8.11	644.5	8.06	586.0	8.05	1077.4
	*Circadian Ka and Ke*	2.93	525.8	3.38	382.6	4.65	476.7	6.71	581.7	7.31	554.8	7.83	1065.6
Hill coefficient (n≠1)	*No circadian Kx*	4.06	573.1	4.19	401.0	13.04	872.9	8.30	651.3	9.25	641.3	9.19	1157.5
	*Circadian Ka*	3.14	533.5	3.74	391.6	6.20	549.0	7.07	596.7	8.16	593.5	8.15	1086.6
	*Circadian Ke*	2.97	525.2	3.74	391.7	6.29	553.4	7.69	626.4	7.98	584.8	8.03	1078.3
	*Circadian Ka and Ke*	**2.71**	**517.6**	**3.28**	**382.0**	**4.32**	**463.3**	**6.48**	**573.2**	**7.03**	**543.6**	**7.75**	**1062.4**

**Table 2:** Objective function (*Fobj*) and corrected Akaike information criterion (*AICc*) values for prodrug models tested.

		Ramipril		Enalapril		Spirapril		Perindopril	
	Models	*Fobj*	*AICc*	*Fobj*	*AICc*	*Fobj*	*AICc*	*Fobj*	*AICc*
No Hill coefficient (n = 1)	*No circadian Kx*	12.08	834.5	4.80	2644.1	7.75	596.5	6.91	672.3
	*Circadian Ka*	11.84	827.7	4.40	2570.2	6.68	549.7	2.95	487.0
	*Circadian Ke*_*1*_	11.80	825.8	4.68	2621.5	6.65	548.2	2.82	480.7
	*Circadian Ke*_*2*_	11.04	789.2	4.70	2628.9	6.62	546.5	2.59	469.5
	*Circadian Km*	10.50	763.2	4.46	2578.6	6.57	544.1	3.24	500.5
	*Circadian Ka and Ke*_*1*_	7.92	644.2	4.40	2571.7	6.29	535.5	2.58	473.9
	*Circadian Ka and Ke*_*2*_	7.92	644.4	4.42	2575.2	**6.15**	**529.2**	2.41	465.9
	*Circadian Ka and Km*	7.97	646.5	4.42	2577.7	6.23	532.6	2.85	486.9
	*Circadian Ke*_*1*_ *and Ke*_*2*_	10.31	758.8	4.64	2617.7	6.54	547.6	2.41	465.9
	*Circadian Ke*_*1*_ *and Km*	8.62	677.8	4.48	2588.8	6.55	548	2.66	477.4
	*Circadian Ke*_*2*_ *and Km*	8.83	688.0	4.44	2580.0	6.37	539.3	2.59	474.2
Hill coefficient (n≠1)	*No circadian Kx*	11.97	831.2	4.52	2589.8	7.72	597.3	6.89	673.7
	*Circadian Ka*	11.67	821.9	4.06	2505.5	6.68	552.1	2.52	468.3
	*Circadian Ke*_*1*_	11.75	825.5	4.36	2564.3	6.65	550.5	2.57	471.0
	*Circadian Ke*_*2*_	11.04	791.7	4.40	2571.6	6.59	547.8	**2.43**	**464.0**
	*Circadian Km*	10.16	749.2	4.08	2507.4	6.41	538.9	2.88	486.0
	*Circadian Ka and Ke*_*1*_	**7.63**	**632.9**	4.02	2501.4	6.20	533.8	2.42	468.6
	*Circadian Ka and Ke*_*2*_	7.92	646.9	**3.98**	**2493.6**	6.11	529.4	2.41	468.4
	*Circadian Ka and Km*	7.68	635.2	4.06	2507.0	6.23	535.1	2.49	471.9
	*Circadian Ke*_*1*_ *and Ke*_*2*_	10.27	759.7	4.34	2564.8	6.38	542.4	2.39	467.0
	*Circadian Ke*_*1*_ *and Km*	8.58	678.7	4.04	2503.4	6.29	538.1	2.55	474.9
	*Circadian Ke*_*2*_ *and Km*	8.51	675.4	4.06	2510.3	6.28	537.5	2.40	467.7

**Fig 2:**
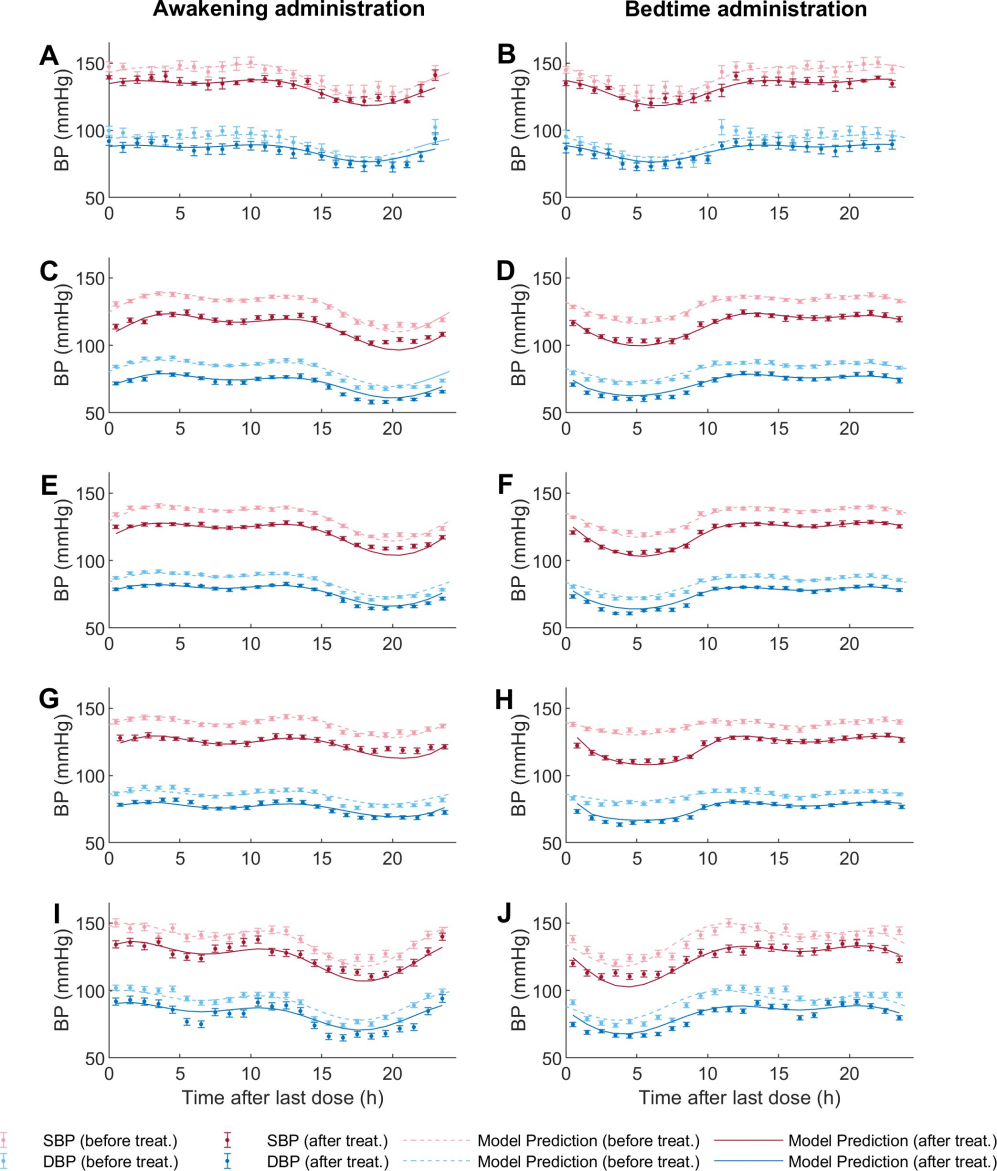
Model fitting to the experimental data for non-prodrug models. The figures on the left correspond to awakening/morning administration and on the right to bedtime/evening administration. Each figure shows data with their respective standard errors (SE) for *SBP* before treatment (light red dots) and *DBP* (light blue dots), and for *SBP* after treatment (dark red dots), *DBP* (dark blue dots). The dotted line represents the model prediction before treatment, and the solid line represents the model prediction of each model after the last dose. Fig 2A and 2B, amlodipine; Fig 2C and 2D, olmesartan; Fig 2E and 2F, telmisartan; Fig 2G and 2H, valsartan; Fig 2I and 2J, lisinopril.

**Fig 3:**
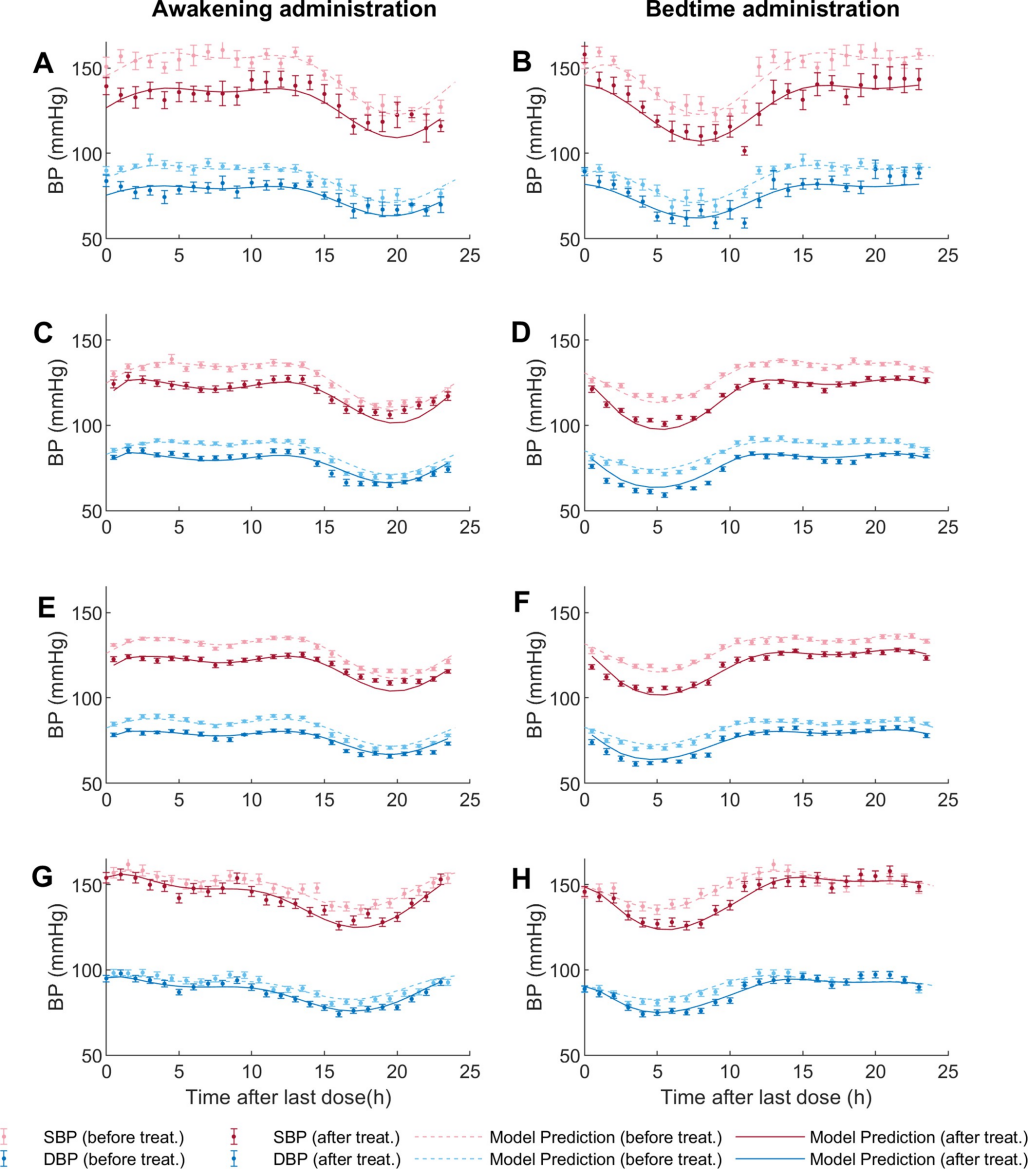
Model fitting to the experimental data for prodrug models. The figures on the left correspond to awakening/morning administration and on the right to bedtime/evening administration. Each figure shows data with their respective standard errors (SE) for *SBP* before treatment (light red dots) and *DBP* (light blue dots), and for *SBP* after treatment (dark red dots), *DBP* (dark blue dots). The dotted line represents the model prediction before treatment, and the solid line represents the model prediction of each model after the last dose. Fig 3A and 3B, enalapril; Fig 3C and 3D, ramipril; Fig 3E and 3F, spirapril; Fig 3G and 3H, perindopril.

Simulations of selected PK-PD models and 24-hours systolic blood pressure (*SBP*) and diastolic blood pressure (*DBP*) data after treatment are shown for awakening and bedtime administration of non-prodrug models in Fig 2 and of prodrug models in Fig 3. All selected non-prodrug models included circadian variation in absorption and elimination kinetic constants through a one-component periodic function. In addition, *AICc* was lower for models with *n* ≠ 1 in the effect equation. Table D in S1 Text shows the estimated parameters of selected non-prodrug models (parameters of Eqs 4–7 and 13) with their respective 95% confidence intervals (*CI*); *CI* units are the same as the corresponding parameter. For the non-prodrug models, *CI* values indicate that all parameters are statistically different from zero, except for *n* and *Ke* from the nifedipine model; therefore, it is not possible to determine whether these parameters are relevant to describe the effect of nifedipine.

Given the different physicochemical properties, routes of metabolism/elimination, and oral and tissue absorption between prodrug ACE inhibitors used in this work, it is not surprising that the models selected for the different antihypertensive prodrugs were also different. The model selected for ramipril included circadian variations in the kinetic constants *Ka* and *Ke*_*1*_, with constant *Km* and *Ke*_*2*_. In contrast, enalapril and spirapril models showed a lower *AICc* when including circadian *Ka* and *Ke*_*2*_. On the other hand, perindopril had a lower *AICc* when including only circadian *Ke*_*2*_. Notably, in all selected non-prodrug models, the elimination constant that presented the lowest mean pharmacokinetic constant baseline (*Ke*_*1*_ *or Ke*_*2*_) was the one dependent on circadian variation. Furthermore, all the medications except spirapril showed a better *AICc* with *n* ≠ 1 (see Table 2). Table E in S1 Text summarizes the estimated parameters of selected prodrug models (parameters of Eqs 8–13) with their respective *CI*, which units are the same as the corresponding parameters. In this case, the *CI* values indicate that all parameters differ statistically from zero.

Simulations including the concentration variable for nifedipine and enalapril are depicted in Figs A and B in S1 Text. The simulation for three administration times of lisinopril is also shown (Fig C in S1 Text) since it is the only medication with data available for more than two times.

### Sensitivity and identifiability analysis

**Fig 4:**
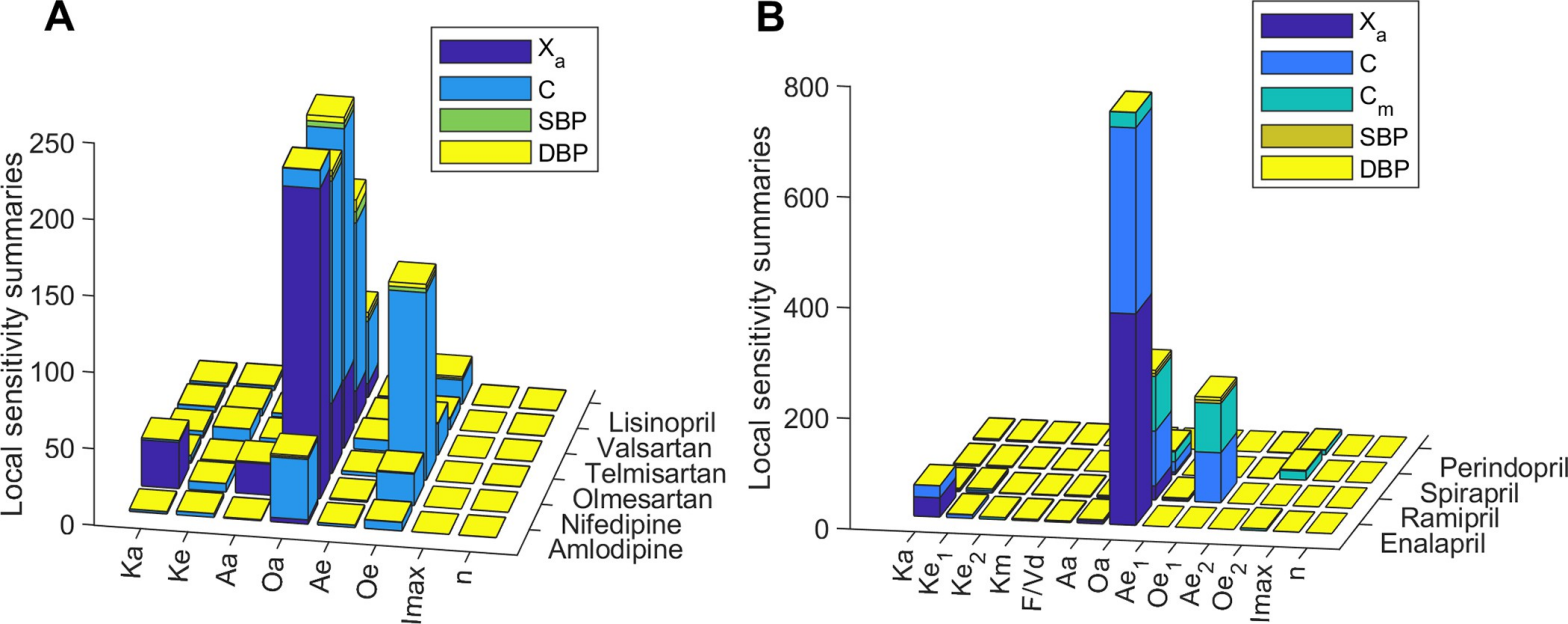
**Local sensitivity summaries for non-prodrug models (A) and prodrug models (B).** Sensitivity values are shown stacked for each model variable (*Xa*: drug available to be absorbed, *C*: drug concentration, *Cm*: metabolite concentration, *SBP*: systolic blood pressure, *DBP*: diastolic blood pressure).

In order to evaluate the relevance of each parameter on the variables of prodrug and non-prodrug models, a local sensitivity analysis was carried out. Local analysis showed high sensitivity of the absorption and elimination kinetic constants on the variables related to circadian behavior. In contrast, parameters *Imax* and *n* have low sensitivity in all models (Fig 4). The parameter *Oa* is the one with the highest sensitivity in all the non-prodrug models, followed by *Oe*, *Ka* and *Aa*. Regarding prodrug models, *Oa* parameter showed the highest sensitivity for the models that include circadian variation of *Ka* (enalapril, ramipril, and spirapril). In contrast, the highest sensitivity for the perindopril model is for *Oe*_*2*_. Other parameters that present high sensitivity are *Ka* for enalapril, *Oe*_*1*_ for ramipril, and *Oe*_*2*_ for spirapril. In summary, higher sensitivities are observed for parameters related to circadian functions of pharmacokinetic constants.

On the other hand, we performed an identifiability analysis for each model to determine if the parameters present a low correlation between them. This analysis revealed different correlation patterns between both non-prodrug and prodrug model parameters (Fig D in S1 Text). Correlation matrices show a high correlation (>0.95) between *n* and *Imax* parameters for telmisartan, olmesartan, lisinopril, and enalapril models. Furthermore, parameters *Ke* and *Ae* are highly correlated in the telmisartan model, as well as parameters *Ka* and *Aa*, and *Ke* and *Oe* in the nifedipine model. In the case of enalapril, there is also a high correlation between *Ke*_*1*_ and *Km*. The ramipril model showed a high correlation between *Ke*_*1*_ and *F/Vd*, and spirapril between parameters *Ke*_*2*_ and *Ae*_*2*_ and between *Km* and *Ae*_*2*_. Finally, the correlation between all the parameters for the amlodipine and valsartan models is less than 0.95. In summary, the highly correlated parameters are n, Imax and periodic function parameters that describe the circadian rhythm of pharmacokinetic processes. The highly correlated parameters of the same non-prodrug model differed between different antihypertensive drugs. That means that the high correlations found in the non-prodrug model are not due to the lack of structural identifiability but to the fitted data. Probably, the same applies to highly correlated parameters of prodrug models. Therefore, to uniquely determine these parameters, it is necessary to have more data, ideally including plasma concentrations for each antihypertensive drug and evaluations at more than two dosing times.

### Dosing time optimization

Using data from untreated dipper and non-dipper hypertensive subjects from Hermida et al. [32], parameters that describe the circadian rhythm of BP (Eqs 1–3) were estimated (Table F and Fig E in S1 Text). PK-PD models and BP parameters for dipper and non-dipper subjects allowed to simulate BP profiles for administration times between 0 and 24 hours, thus calculating optimization objectives.

**Fig 5:**
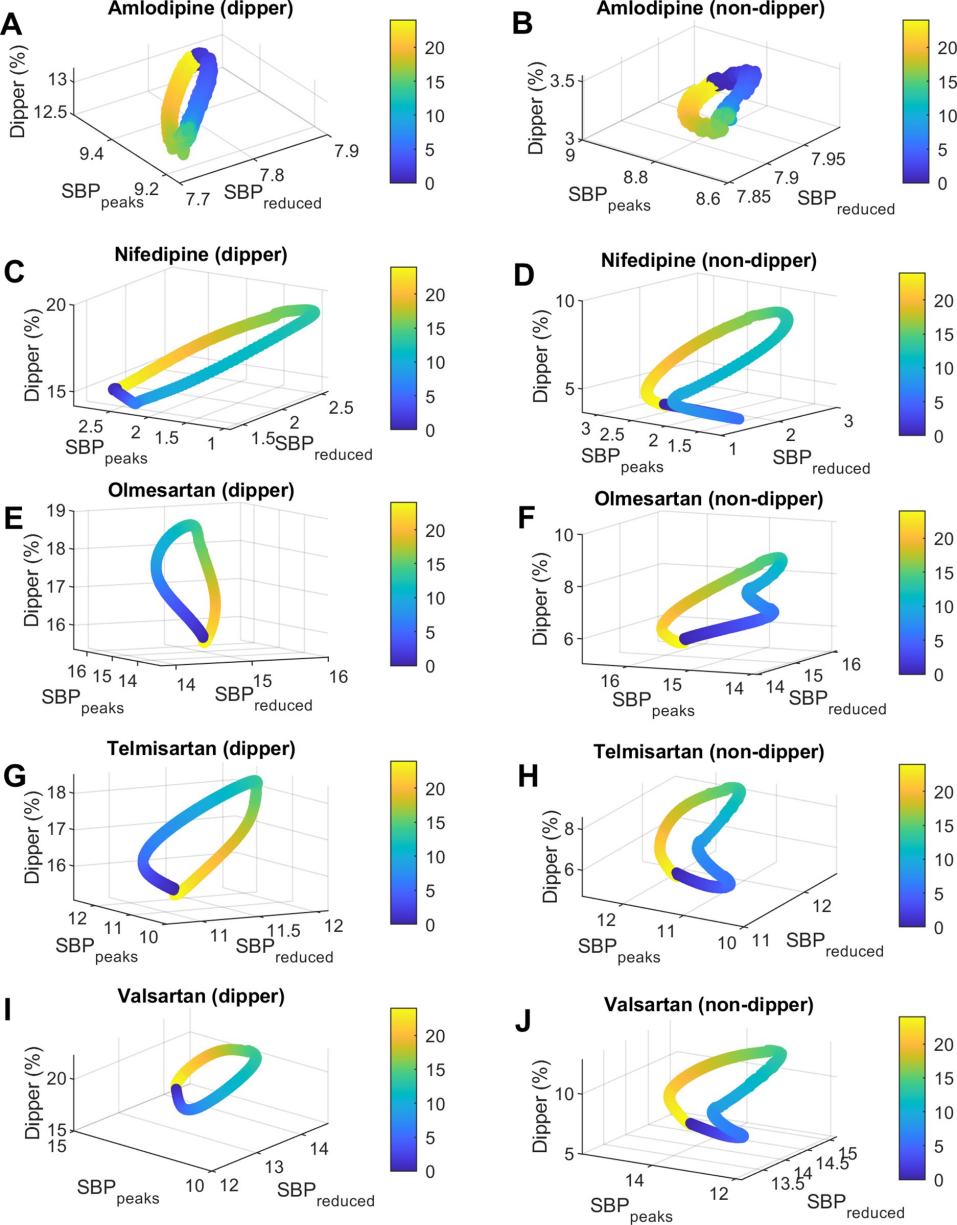
Relationship between dosing time and *SBP* dipper percentage, average *SBP* reduction (*SBP*_*reduced*_), and average *SBP* peaks reduction (*SBP*_*peaks*_) for amlodipine, nifedipine, telmisartan, olmesartan, and valsartan. The figures on the left show the results for dipper subjects and those on the right for non-dipper. Fig 5A and 5B, amlodipine; Fig 5C and 5D, nifedipine; Fig 5E and 5F, olmesartan; Fig 5G and 5H, telmisartan; Fig 5I and 5J, valsartan. Dosing time (*Ta*) is represented in colors from 0 to 24 hours after awakening (colorbar).

**Fig 6:**
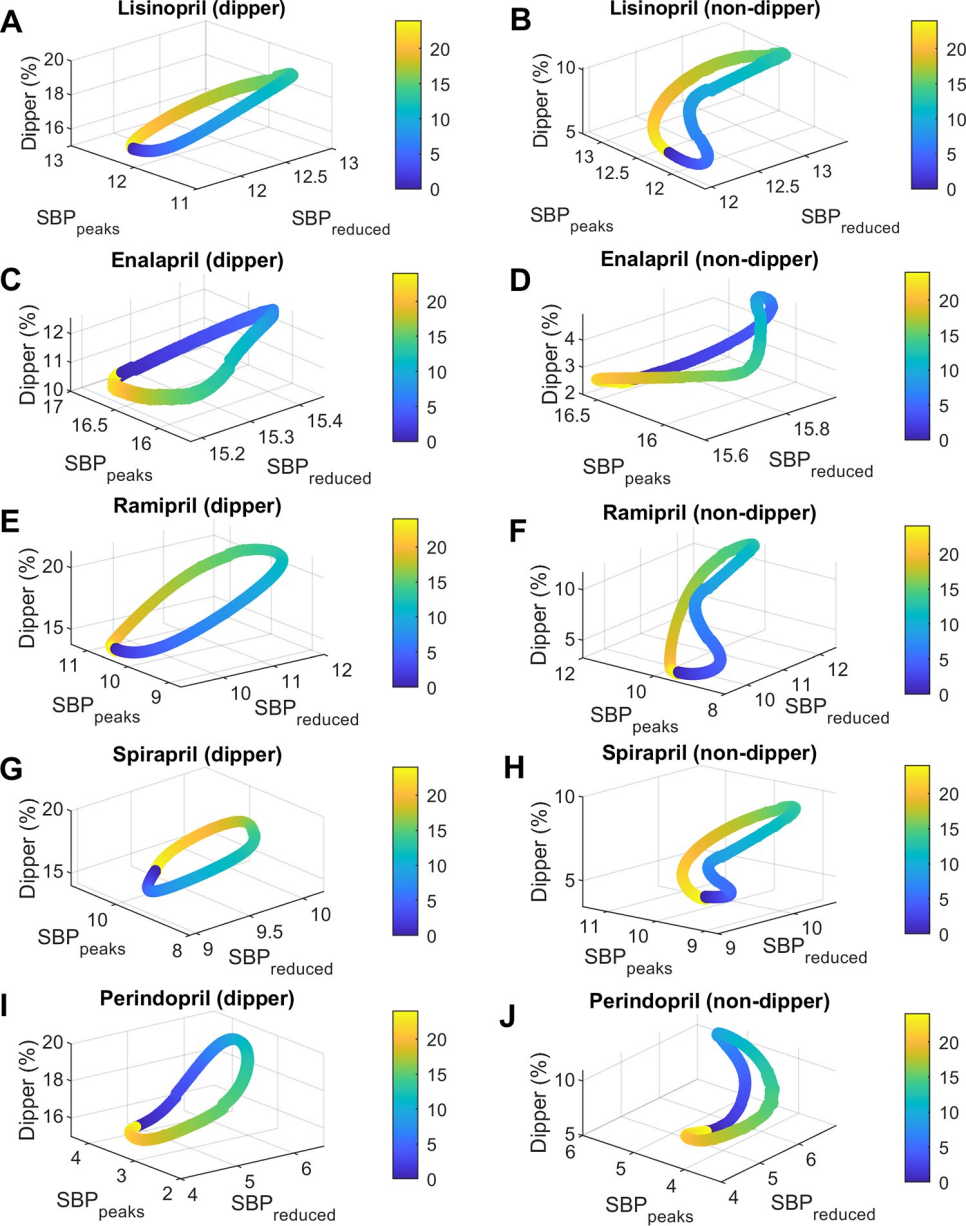
Relationship between dosing time and *SBP* dipper percentage, average *SBP* reduction (*SBP*_*reduced*_), and average *SBP* peaks reduction (*SBP*_*peaks*_) for lisinopril, enalapril, ramipril, spirapril and perindopril. The figures on the left show the results for dipper subjects and those on the right for non-dipper. Fig 6A and 6B, lisinopril; Fig 6C and 6D, enalapril; Fig 6E and 6F, ramipril; Fig 6G and 6H, spirapril; Fig 6I and 6J, perindopril. Dosing time (***Ta***) is represented in colors from 0 to 24 hours after awakening (colorbar).

The relationship between administration time (*Ta*) and the three objectives (dipper percentage, average *SBP* reduction (*SBP*_*reduced*_), and average *SBP* peaks reduction (*SBP*_*peaks*_)) is represented in Figs 5 and 6. Note that in all figures and tables, the administration times (*Ta*) are reported as time after awakening (at awakening *Ta* = 0). This allows comparison between studies that had different chronological times of activity/sleep (sleep time occurs between 15–24 hours after awakening approximately). Left panels show simulations for dipper subjects and right panels for non-dipper subjects. Moreover, each row corresponds to a different medication. In general, the patterns vary between different medications and also change between dipper vs. non-dipper profiles. Similar patterns are observed in the case of DBP, the main difference with SBP being the amount of mmHg decreased of *DBP*_*reduced*_ and *DBP*_*peaks*_ (see Figs F and G in S1 Text).

Dosing times (*Ta*) for maximum and minimum values of dipper percentage, average reduction (*BP*_*reduced*_), and average peaks reduction (*BP*_*peaks*_) of *SBP* and *DBP* for dipper subjects are shown in Tables G and H in S1 Text, respectively. The same results for non-dipper subjects are shown in Tables I and J in S1 Text. Tables G-J in S1 Text summarize the difference between the maximum and minimum values reached for each target metric (dipper percentage, *ΔDip*; average reduction, *ΔSBP*_*reduced*_ and *ΔDBP*_*reduced*_; and average peaks reduction, *ΔSBP*_*peaks*_ *and ΔDBP*_*peaks*_) by varying dosing times.

For dipper subjects, dipper percentages were optimal for all drug models; only ramipril and valsartan presented values greater than 20% in a limited range of *Ta*. Meanwhile, for non-dipper subjects, models predicted low dipper values for amlodipine and enalapril for all *Ta* (maximum values reached: 3.6% and 4.9%, respectively). Suboptimal values of dipper percentage (between 8.5% - 9.7%) were reached at specific administration times for nifedipine, spirapril, telmisartan and olmesartan. However, optimal values within a particular range of *Ta* were obtained for lisinopril, ramipril, perindopril and valsartan. For both dipper and non-dipper patterns, minimum values of dipper percentage were obtained for administrations close to waking time. Conversely, maximum values were obtained in the afternoon for enalapril and perindopril (9–10 hours after awakening) and close to bedtime for the other medications (12.5–14.5 hours after awakening). Higher *ΔDip* values were obtained for ramipril, spirapril and valsartan for both dipper and non-dipper subjects.

Regarding the other objectives, *BP*_*reduced*_ values were lowest for nifedipine and perindopril (1.3–7 mmHg for *SBP* and 0.8–4.3 mmHg for *DBP*) and highest for olmesartan and enalapril (13.8–16.1 mmHg for *SBP* and 8.7–10 mmHg for *DBP*). Lower *BP*_*reduced*_ values for both dipper and non-dipper subjects were obtained by administering ramipril and lisinopril after awakening (3–4 hours after awakening); spirapril, nifedipine, telmisartan, olmesartan, and valsartan in the afternoon (5–9 hours after awakening); and amlodipine, perindopril, and enalapril during sleep (16–21.5 hours after awakening). Higher *BP*_*reduced*_ values were obtained before awakening for spirapril, telmisartan, valsartan and olmesartan (17–21.5 hours after awakening approximately); after awakening for amlodipine (1–2 hours after awakening); later times for perindopril and enalapril (6–8 hours after awakening); and at bedtime for nifedipine, lisinopril, ramipril (14–15.5 hours after awakening). On the other hand, the reduction of the peaks (*BP*_*peaks*_) was lower for nifedipine and perindopril (0.9–5.7 mmHg for *SBP* and 0.5–3.6 mmHg for *DBP*) and higher for enalapril and olmesartan (13–16.9 mmHg for *SBP* and 8.3–10.8 mmHg for *DBP*). Dipper subjects obtained minimum *BP*_*peaks*_ values in the afternoon for telmisartan and ramipril (8–9 hours after awakening) and close to bedtime for the other medications (~11–15 hours after awakening). In contrast, minimum values of *BP*_*peaks*_ for non-dipper subjects were obtained some hours after awakening for nifedipine, lisinopril, ramipril, telmisartan and valsartan (4–5.5 hours after awakening); and close to bedtime for spirapril, olmesartan, amlodipine, enalapril and perindopril (11.5–15.5 hours after awakening). Maximum *BP*_*peaks*_ values were also reached at different times between dipper and non-dipper subjects. For dipper subjects, maximum *BP*_*peak*_ values were obtained close to awakening for amlodipine, nifedipine and enalapril; some hours after awakening for perindopril (~6 hours after awakening); and during the night rest for lisinopril, ramipril, spirapril, olmesartan, valsartan and telmisartan (16.5–23 hours after awakening). In comparison, non-dipper subjects had higher *BP*_*peaks*_ when administering ramipril, lisinopril, telmisartan, spirapril, valsartan, olmesartan, nifedipine and enalapril during the sleep time (16–22 hours after awakening); amlodipine close to awakening; and perindopril later (~8 hours after awakening). Higher *ΔSBP*_*reduced*_ and *ΔDBP*_*reduced*_ values were obtained for ramipril, perindopril and valsartan for both dipper and non-dipper subjects. In contrast, higher *ΔSBP*_*peaks*_ and *ΔDBP*_*peaks*_ values were obtained for ramipril, spirapril, olmesartan and valsartan for both dipper and non-dipper subjects.

In order to find optimal administration times for each medication and BP profile type, the results obtained for all possible *Ta* parameters were evaluated and those reaching the current therapeutic goal of BP <130/80 and, at the same time, optimal values of dipper percentage were selected. Ranges of maximum and minimum *SBP* values (*SBP*_*min*_ and *SBP*_*max*_), *DBP* (*DBP*_*min*_ and *DBP*_*max*_), and dipper percentage, as well as the *Ta* in which the objectives BP<130/80 mmHg and a dip of 10–20% are reached, are shown in Table 3 for dipper subjects and Table 4 for non-dipper subjects. In addition, both tables show a column that indicates the optimal administration time for each antihypertensive medication based on an activity/rest cycle between 8 a.m. (upon awakening) and 11 p.m. (bedtime).

**Table 3:** Predicted results of BP and optimal dosing times (Ta) after awakening using PK-PD models at different dosing times for dipper subjects.

Medications	*SBP*_*min*_ (mmHg)	*DBP*_*min*_ (mmHg)	*SBP*_*max*_ (mmHg)	*DBP*_*max*_ (mmHg)	*Ta* (hh:mm) for BP<130/80 mmHg	*% Dipper*	*Ta* (hh:mm) for Dip 10–20%	*Optimal time of day* ***
Amlodipine	101.6–102.1	64.0–64.3	128.1–128.6	81.6–81.9	00:04*	12.6–13.2	0–24	morning (upon waking)
Nifedipine	101.8–106.5	64.2–67.1	136.9–137.8	87.1–87.7	03:16** and 02:54*	14.2–18.9	0–24	~ 3 h after awakening
Lisinopril	91.6–96.7	57.7–60.9	125.0–129.6	79.5–82.5	18:16–00:49	15.3–19.5	0–24	morning (upon waking)
Enalapril	92.7–95.3	58.4–60.1	120.3–122.6	76.5–78.1	0–24	10.1–12.5	0–24	any time
Ramipril	91.3–99.2	57.5–62.5	126.8–135.7	80.9–86.5	12:21–00:09^Δ^ and 16:18*	13.8–21.3	14:31–9:19	bedtime or upon waking
Perindopril	96.0–101.6	60.5–64.1	133.4–137.3	84.8–87.4	01:44** and 01:37*	15.2–19.8	0–24	1–2 hours after waking up
Spirapril	94.0–100	59.0–63.0	126.3–132.3	80.2–84.2	13:35–01:34^Δ^ and 0:02*	14.0–19.4	0–24	morning (upon waking)
Telmisartan	93.8–97.1	59.0–61.2	125–131.3	79.6–83.7	16:59–00:28	15.1–18.5	0–24	morning (upon waking)
Olmesartan	91.3–94.0	57.4–59.3	122.4–129	78.0–82.3	14:36–01:48	15.4–19	0–24	bedtime or upon waking
Valsartan	88.9–95.0	55.8–59.9	124.2–132.5	79.2–84.5	18:36–00:55	16.0–22.3	17:18–8:31	morning (upon waking)

* *Ta* after awakening in which the minimum *DBP*_*max*_ value is reached (values less than 80 mmHg are not obtained).

** *Ta* after awakening in which the minimum *SBP*_*max*_ value is reached (values less than 130 mmHg are not obtained).

^Δ^ *Ta* after awakening range in which *SBP* predicted values are less than 130 mmHg.

*** Optimal time of day assuming an activity/rest cycle between 8 am (upon awakening) and 11 pm (bedtime).

**Table 4:** Predicted results of BP and optimal dosing times (Ta) after awakening using PK-PD models at different dosing times for non-dipper subjects.

Medications	*SBP*_*min*_ (mmHg)	*DBP*_*min*_ (mmHg)	*SBP*_*max*_ (mmHg)	*DBP*_*max*_ (mmHg)	*Ta* (hh:mm) for BP<130/80 mmHg	*% Dipper*	*Ta* (hh:mm) for Dip 10–20%	*Optimal time of day* ***
Amlodipine	116.8–117.3	72–72.3	128.5–129	79.6–79.9	0–24	3.1–3.6	14:24 for 3.6%	late evening
Nifedipine	117.0–122.3	72.1–75.4	134.7–137.5	83.3–85.1	0:36** and 0:20*	3.7–8.9	13:31 for 8.9%	not recommended
Lisinopril	105.3–111.2	64.8–68.5	123–127.8	76.2–79.2	0–24	5.5–10.1	12:18–13:54	evening
Enalapril	106.5–109.4	65.6–67.4	119.6–122.6	74–76	0–24	2.4–4.9	9:30 for 4.9%	late afternoon
Ramipril	104.9–113.9	64.6–70.2	126.4–135.2	78.5–83.8	11:56–18:29	3.2–11.7	9:13–14:48	late evening
Perindopril	110.3–116.9	67.9–72	131.4–136.3	81.3–84.6	0:10** and 00:10*	5.9–11.0	8:49–14:24	late afternoon
Spirapril	108.0–114.7	66.3–70.7	124.4–131.1	77–81.2	10:04–23:55	3.5–9.6	13:37 for 9.6%	late evening
Telmisartan	107.9–111.6	66.4–68.8	125–131.1	77.5–81.4	9:30–0:20	4.7–8.6	13:25 for 8.6%	late evening
Olmesartan	104.9–108	64.5–66.6	120.8–127.3	74.8–78.9	0–24	5.0–9.1	13:25 for 9.1%	late evening
Valsartan	102.1–109	62.7–67.2	122.9–131.2	76.3–81.5	10:51–0:59	5.8–12.9	8:07–17:42	late evening or bedtime

*Ta after awakening in which the minimum *DBP*_*max*_ value is reached (values less than 80 mmHg are not obtained).

**Ta after awakening in which the minimum *SBP*_*max*_ value is reached (values less than 130 mmHg are not obtained).

***Optimal time of day assuming an activity/rest cycle between 8 am (upon awakening) and 11 pm (bedtime).

In the case of dipper subjects, predicted results show that lisinopril, enalapril, telmisartan, valsartan and olmesartan allow reaching BP<130/80 mmHg and, at the same time, optimal dipper percentage values. Optimal *Ta* parameters were obtained at awakening for lisinopril, telmisartan, valsartan, and olmesartan (administration at bedtime is also optimal for olmesartan), and at any *Ta* for enalapril. Amlodipine, ramipril and spirapril models predict a maximum *DBP* slightly higher than the limit of 80 mmHg, with optimal *Ta* upon awakening for amlodipine and spirapril, and at bedtime for ramipril (but the optimal *Ta* of ramipril for *SBP* was upon awakening). In contrast, nifedipine and perindopril show suboptimal results, not obtaining a reduction in *SBP* and *DBP* enough to reach the therapeutic objective BP<130/80 mmHg at any *Ta*. Despite the latter, administration near awakening had a greater effect for both nifedipine and perindopril in dipper subjects.

For non-dipper subjects, results showed that reaching both goals at the same time (BP<130/80 and dipper percentage of 10–20%) is more difficult than for dipper subjects. Only lisinopril, ramipril and valsartan models predicted optimal results. Optimal *Ta* for lisinopril is in the range of 12.5–14.0 hours after awakening, for ramipril between 9–15 hours after awakening and valsartan between 8–18 hours after awakening. Suboptimal values of dipper percentage (8.5–9.6%), but optimal values of BP were obtained when administering spirapril, telmisartan and olmesartan some hours before bedtime (13–14 hours after awakening). Lower dipper percentage values (3.5–4.9%), but optimal BP values were obtained when administering enalapril and amlodipine ~9.5 and ~14.5 hours after awakening, respectively. Finally, nifedipine and perindopril showed the worst results for dipper subjects. In the case of nifedipine, neither of the two objectives was achieved; it showed the highest percentage of dipper (~ 8.9%) at ~13.5 hours after awakening and greater effects on BP when administered after awakening. On the other hand, perindopril achieved optimal dipper percentage values for *Ta* some hours before bedtime (approximately 8.5–14.5 hours after awakening) but a greater effect on BP for *Ta* upon awakening.

In summary, dipper subjects had better outcomes when taking antihypertensive medications at awakening, except for ramipril. At the same time, non-dipper subjects models predicted better results close to bedtime administration, except for enalapril and perindopril. Perindopril and nifedipine had more difficulties achieving therapeutic goals, mainly in non-dipper subjects. Therefore, optimal administration times differed between antihypertensive medications and BP profiles.

## Discussion

Usually, administration upon awakening of antihypertensive medications is recommended to reduce daytime BP surges. However, in recent years, several studies have shown the benefits of bedtime/evening administration of these medications [26,31,33,34], mainly due to a more significant effect on BP during night rest and a possible greater effect on morning surge [35]. Variations in the effect of antihypertensive medications when administered at different dosing times have been explained by circadian pharmacokinetic and pharmacodynamic variations, which result in non-intuitive variations in the effect of BP [12,33]. To understand these variations and find the optimal administration times (both to maintain a dipper profile and BP levels <130/80 mmHg) we developed PK-PD models that include the circadian rhythm for antihypertensive non-prodrugs and prodrugs. Using previously published data from healthy subjects or subjects diagnosed with grade I or II AH, non-prodrug and prodrug PK-PD model parameters were estimated. For each antihypertensive medication, models with and without kinetic constants with circadian components were tested and selected based on the corrected Akaike Information Criterion (*AICc*). The *AICc* was established as a selection criterion due to its better ability to identify models with good prediction compared to Bayesian Information Criterion (BIC) and at the same time less overfitting to the data compared to Akaike Information Criterion [36]. Although there is a high correlation between some kinetic parameters (mainly in non-prodrug models), the values of BIC indicate the selection of the same models as *AICc* or models with the same circadian components but without the coefficient *n* on the effect equation (BIC values, data and computational codes for each antihypertensive medication can be reached at https://www.synapse.org/#!Synapse:syn36744682/files/). In addition, sensitivity analysis showed higher sensitivities of the circadian parameters for all the models. Therefore, the incorporation of circadian components on kinetic constants in PK-PD models is consistent and suggests considering these variations in the study of the effect of orally administered antihypertensive medications.

Variations in the selected models, as well as in the sensitivity of the estimated PK parameters of the prodrug models, can be explained by differences in tissue accumulation, elimination/metabolism routes, oral and tissue absorption, and penetration of these drugs [37]. The only prodrug model that did not include the circadian rhythm of *Ka* was perindopril, which has the highest bioavailability among the prodrugs studied (95% versus 50–60%, see Table C in S1 Text). In contrast, enalapril, perindopril and spirapril models included the circadian rhythm of *Ke*_*2*_ and only ramipril included that of *Ke*_*1*_, which is explained by their different elimination routes and rates. Renal clearance and half-life times of enalapril, perindopril, and spirapril indicate that their elimination rate is greater than their active metabolite, enalaprilat, perindoprilat, and spiraprilat, respectively [38–41]. In contrast, ramiprilat is eliminated at a greater rate than its parent drug, ramipril [42]. This supports the higher estimated values of *Ke*_*1*_ relative to *Ke*_*2*_ for enalapril, perindopril, and spirapril, and the opposite for ramipril (*Ke*_*2*_ > *Ke*_*1*_). Consequently, elimination constants with the lowest estimated value, that is, those that describe a slower process, are selected as dependent on the circadian rhythm for each prodrug.

Regarding PD parameters, a low sensitivity was obtained for *n* and *Imax* of selected models. This can be explained by how the sensitivity was calculated since normalized sensitivity summaries were estimated for each variable. Namely, since *SBP* and *DBP* profiles change in low percentages with respect to their respective baseline values, normalized sensitivity values for these variables are lower than the sensitivity of the *C*, *Cm* or *Xa* variable parameters. But, if we analyze the effect on BP, parameters are relevant to achieve a good prediction, which is supported by the obtained *AICc* Value.

Although concentration data are not available for all antihypertensive medications, the identifiability analysis shows that the PK parameters can be reasonably estimated in most cases. This is because the PK-PD models assume that changes in the effect on BP can be explained by changes in pharmacodynamics or pharmacokinetic processes. The *SBP* and *DBP* equations show that the circadian changes in pharmacodynamics are directly related to the input function *k*_*in*_*(t)* (Eq 3), which parameters are set before estimating the circadian pharmacokinetic parameters. Therefore, circadian changes in BP effect that are not explained by *k*_*in*_*(t)* must necessarily be explained by circadian changes in pharmacokinetic parameters (i.e., changes in plasma concentration). In addition, we show that, for the same non-prodrug model, highly correlated parameters were different for different antihypertensive medications (Fig D in S1 Text), so the high correlation between some parameters is not due to structural lack of identifiability but rather to the insufficient information provided by the adjusted data. However, in the case of prodrug models, estimating highly correlated pharmacokinetic parameters probably requires larger data sets, including concentrations of the drug and the active metabolite for more than two dosing times. Therefore, future clinical studies of antihypertensive medications that include plasma concentration data for more than two dosing times are recommended to estimate these parameters. Moreover, a personalized clinical study would make it possible to extend the subpopulation analysis of dipper and non-dipper subjects performed in this work to individual-level information, considering both pharmacokinetic parameters and individual BP profiles and thus estimating the optimal time and dose for each patient.

Subsequently, the developed models were used to optimize the dosing time of dipper and non-dipper subjects. The latter was carried out by replacing parameters of Eqs 1–3 with parameters obtained by fitting data from untreated dipper and non-dipper subjects from Hermida et al [32]. Replacing previously estimated BP parameters and still using the same PK-PD parameters could be questionable because underlying conditions or comorbidities such as diabetes or adrenal insufficiency and different lifestyles can generate different BP patterns with altered regulatory mechanisms and, therefore, biased results of antihypertensive medication responses. However, our methodology is supported by the fact that study subjects met the same inclusion and exclusion criteria, so PK parameters should not be statistically different. Furthermore, different doses and *IC*_*50*_ values between dipper and non-dipper subjects have not been established so far. Nevertheless, a clinical study with personalized effect data from dipper versus non-dipper patients for each antihypertensive medication could provide further insight, mainly due to possible variations in dose-response curves between patients with different BP profiles.

Maximum reduction on BP peaks (*BP*_*peaks*_) was also reached at different times between dipper and non-dipper subjects. For dipper subjects, maximum *BP*_*peaks*_ were obtained close to awakening time for amlodipine, nifedipine and enalapril; some hours after awakening for perindopril (~6 hours after awakening); and during sleep for lisinopril, ramipril, spirapril, olmesartan, valsartan and telmisartan (16.5–23 hours after awakening). In comparison, non-dipper subjects have a higher reduction in BP peaks when administering ramipril, lisinopril, telmisartan, spirapril, valsartan, olmesartan, nifedipine, and enalapril during sleep time (16–22 hours after awakening); amlodipine close to awakening; and perindopril later (~8 hours after awakening).

To understand the relationship between administration time and different therapeutic goals (dipper percentage, *BP*_*reduced*_ and *BP*_*peaks*_), we evaluated the PK-PD models developed with administration times from 0 to 24 hours. Results show that maximum values of dipper percentage, *BP*_*reduced*_ and *BP*_*peaks*_ occur at different dosing times (competing optimization objectives). Furthermore, patterns of relationship between dipper percentage, *BP*_*reduced*_, and *BP*_*peaks*_ differ between medications and predicted outcomes for dipper and non-dipper subjects. Therefore, optimal *Ta* parameters are not the same for all cases. On the one hand, dipper percentage is higher when AH medications are administered close to bedtime except for enalapril and perindopril. On the other hand, *BP*_*reduced*_ is higher for administration times close to awakening for olmesartan and amlodipine, later times for enalapril and perindopril, and administration at bedtime for the other medications, both for dipper and non-dipper subjects. Slight differences were found between dipper and non-dipper subjects regarding *BP*_*peaks*_. This is probably due to the greater distance between average BP values and BP peaks in dipper patterns during the day. For dipper subjects, some medications obtain higher *BP*_*peaks*_ values when administered close to awakening. In comparison, for non-dipper subjects, more medications reach higher values when simulating bedtime or during sleep administration. Still, for both types of subjects, the best results are obtained at administration times less feasible in practice (during sleep).

As mentioned in the introduction, the optimization of the therapeutic objectives evaluated (dipper percentage, *BP*_*reduced*_ and *BP*_*peaks*_) has been associated with lower long- and short-term cardiovascular risk [26–28]. Indeed, decreased BP during sleep is shown to have the greatest impact on long-term cardiovascular risk [6]. However, there is no quantitative estimate of how each of these therapeutic targets affects cardiovascular risk. Therefore, it is difficult to define multi-objective optimization weights to find an optimal administration time for each type of subject and drug. In addition, our results indicate that the times when higher dipper percentages are obtained coincide with those in which a more significant BP decrease during sleep is obtained (non-competitive optimization objectives); therefore, only the dipper percentage was included in the analyses. That is why the criteria used in this study to obtain optimal administration times are based on the therapeutic objective defined by the current American Clinical Guidelines (<130/80 mmHg) and to obtain a dipper pattern (dip 10–20%).

The results indicate that for dipper subjects, better outcomes would be obtained by administering medications close to awakening, except for ramipril and olmesartan, which can be taken either upon awakening or at bedtime, and enalapril at any time. Indeed, bedtime administration of ramipril and valsartan in dipper subjects could generate nocturnal hypotension, so morning administration is recommended to avoid possible adverse effects during night rest. Furthermore, administration in the morning is also recommended for subjects with nocturnal hypotension to prevent significant complications.

For non-dipper subjects the best results are obtained for evening/bedtime dosing times, except for enalapril and perindopril, which show better results when administered in the afternoon, and only nifedipine failed to reach both dipper percentage and target BP. The greater demand for the effect during night rest made finding optimal Ta parameters for these subjects more challenging. This could be overcome by administering the antihypertensive medication twice daily or by giving a combination of drugs, so both alternatives should be tested in a clinical trial. The main difficulties were observed for perindopril and nifedipine. Perindopril allows a greater nocturnal reduction in BP with administration at bedtime, as reported in [43]; however, the times when BP values <130/80 mmHg are reached do not coincide with the greater nocturnal reduction in the case of non-dippers. Nifedipine even failed to achieve the therapeutic objective for dipper subjects due to its immediate-release formulation, which is why it is not usually used in the chronic treatment of arterial hypertension but rather in hypertensive crises [44]. On the other hand, the drug that presented a smaller variation of effect on BP and dipper percentage was amlodipine, which coincides with the nonsignificant differences between different dosing times observed by other authors [45–48]. Finally, studies of lisinopril, ramipril, enalapril, spirapril, valsartan, telmisartan and olmesartan showed a significant improvement in the dipper percentage in bedtime administration, which is consistent with our analyses [49–61].

In summary, we established different optimal administration times by evaluating the effect on dipper percentage, *BP*_*reduced*_ and *BP*_*peaks*_ and found time intervals when the dipper percentage lays in an optimal range and BP achieves the current therapeutic goal for most of the medications studied. However, a clinical study is necessary to verify the predictions and evaluate the significance of optimizing the dosing time in a personalized way on the effect on BP and the long-term cardiovascular risk.

## Materials and methods

### Data search and selection

A data search was performed to find BP and plasma concentration data of first-line antihypertensive medications for at least two different dosing times. Selected data include healthy subjects or diagnosed subjects with grade I or II AH and people >18 years older. Moreover, studies involving shift-workers, pregnant women, consumers of > 80 g/day of alcohol or > 20 cigarettes/day, high-performance athletes, subjects with severe AH (grade 3, BP ≥180 / 110 mm Hg), or secondary hypertension, and subjects with complex cardiovascular diseases were excluded.

Only PK-PD publications with BP profiles were considered (data presenting only variations or averages were discarded). In all selected studies, antihypertensive medications were administered once daily. PK and PD data were found for enalapril (10 mg at 7 a.m. or 7 p.m.) and nifedipine (10 mg at 8 a.m. or 7 p.m.) from [55,62]. PD data for ramipril (5 mg either on awakening or bedtime), spirapril (6 mg, either on awakening or bedtime), perindopril (4 mg at 9 a.m. or 9 p.m.), lisinopril (20 mg at 8 a.m., 4 p.m. or 10 p.m.), amlodipine (5 mg at 8 a.m. or 8 p.m.), valsartan (160 mg either on awakening or bedtime), olmesartan (20 mg either on awakening or bedtime) and telmisartan (80 mg either on awakening or bedtime) were extracted from [43,47,49,51,52,54,58,63]. For published data in which no chronological time was indicated, wake-up time was established at 8 a.m. and sleep time at 11 p.m., keeping the reported sleep duration (9 hrs). Regarding activity/rest cycle and naps, the nifedipine and enalapril data were obtained by establishing the same cycle of activity/rest, suggesting following the same routines; the lisinopril study established chronological times for activity/rest cycle; the studies of amlodipine, ramipril, spirapril, telmisartan, olmesartan and valsartan, suggested following the same routines and avoiding daytime napping; and patients of the perindopril study were instructed to report their times of going to bed and arising when the BP monitor was in use, and reported times did not indicate naps.

Average experimental data of each antihypertensive medication study and their corresponding standard errors were used both for the estimation of parameters and the subsequent model selection. For BP data without standard error or standard deviation (SD), an approximation of 2% of the average values was used as SE, which is roughly the mean SE of the other data sets. Subsequently, fixed parameters of the PK-PD models (volume of distribution (*Vd*), bioavailability (*F*), and *IC*_*50*_) were set according to respective published information (see Table C in S1 Text) [64–74].

### PK-PD modeling

The PK-PD models were developed using differential equation systems that couple equations from a one-compartment pharmacokinetic model and two indirect effect type I models with periodic production rates for *SBP* and *DBP*, respectively [30].

Firstly, parameters for *SBP* and *DBP* equations were estimated using before-treatment data for each medication (Eqs 1–2). BP differential equations include production (*k*_*in*_) and output constants (*k*_*out*_). But, in order to incorporate the circadian rhythm of BP, the production constant was included as a periodic function in both equations (Eq 3).


$$\frac{dSBP}{dt}=k_{in}(t)-k_{out1}*SBP$$ (1)

$$\frac{dDBP}{dt}=k_{in}(t)-k_{out2}*DBP$$ (2)

$$k_{in}(t)=M+A_{12}\cos\left(W_{12}(t+Ta)+O_{12}\right)+A_{24}cos(W_{24}(t+Ta)+O_{24})$$ (3)

Overall, Eqs 1–3 describe the baseline BP before treatment; where the periodic production rate constant *k*_*in*_*(t)* describes the two-component circadian rhythm of BP and depends on the integration time (*t*, from 0 to 24 hours) and the parameters *M* (mean BP baseline or mesor), *A*_*12*_ (amplitude for 12 hours component), *A*_*24*_ (amplitude for 24 hours component), *W*_*12*_ (angular frequency, 2π/12), *W*_*24*_ (angular frequency, 2π/24), *O*_*12*_ (BP peak time or acrophase for 12 hours component), *O*_*24*_ (BP peak time or acrophase for 24 hours component) and administration time (*Ta*).

Subsequently, pharmacokinetic differential equations and a Hill equation were incorporated on *SBP* and *DBP* differential equations to capture the treatment effect. In the case of non-prodrugs, the models were composed of four ordinary differential equations (Eqs 4–7). The first one describes the quantity available to be absorbed (*Xa*), which initial condition corresponds to the administered dose (ug); the second one represents the plasma concentration of the drug (*C*); and the third and fourth equations to *SBP* and *DBP* after treatment. Thus, the type I effect model of each antihypertensive medication was completed, and Hill coefficient *n* ≠ 1 and *n* = 1 were tested (Eqs 6–7) [75].


$$\frac{dX_{a}}{dt}=-K_{a}(t)*X_{a}$$ (4)

$$\frac{dC}{dt}=\frac{F}{Vd}*X_{a}*K_{a}(t)-K_{e}(t)*C$$ (5)

$$\frac{dSBP}{dt}=k_{in}(t)*\left(1-\frac{I_{max}*C^{n}}{{IC}_{50}^{n}+C^{n}}\right)-k_{out1}*SBP$$ (6)

$$\frac{dDBP}{dt}=k_{in}(t)*\left(1-\frac{I_{max}*C^{n}}{{IC}_{50}^{n}+C^{n}}\right)-k_{out2}*DBP$$ (7)

Eq 4 describes the antihypertensive medication’s absorption rate, where *Xa* corresponds to the amount available to be absorbed, and *Ka(t)* is the absorption constant. Eq 5, which describes the change in plasma concentration over time, is defined by the parameters *F* (bioavailability), *Vd* (volume of distribution), *Ka(t)* and *Ke(t)* that correspond to absorption and elimination constants, respectively. In the *SBP* and *DBP* equations, *n* is the hill coefficient, *IC*_*50*_ is the concentration that produces 50% inhibition, and *Imax* is the maximum inhibitory effect that can be achieved by the antihypertensive medication, which is defined between 0 and 1. *Imax* should not be less than 0 because this would imply increases in BP after administering the antihypertensive medication (opposite effect), and it should be less than 1, since if *Imax* > 1, then high concentrations of the drug could generate BP values close to zero or even negative, which is infeasible due to physiological compensation mechanisms. Moreover, reported *IC*_*50*_ values correspond to the drug concentration that inhibits smooth muscle contraction by 50%, in the case of ARBs and CCBs, and the concentration that inhibits the activity of the angiotensin converting enzyme by 50%, in the case of ACE inhibitors. Therefore, they do not necessarily correspond to concentrations at which a 50% decrease in BP is achieved.

The non-prodrug model was fitted to the amlodipine, nifedipine, lisinopril, valsartan, telmisartan and olmesartan data (although olmesartan medoxomil is a prodrug, for modeling purposes it was considered a non-prodrug due to its rapid bioactivation by hydrolysis [76]).

In the case of prodrugs (enalapril, ramipril, perindopril and spirapril), it was necessary to include an equation for the concentration of the drug and its active metabolite. Therefore, the model for prodrugs corresponds to a system of five ordinary differential equations (Eqs 8 – 12). The first one describes the quantity available to be absorbed (*Xa*), the second one is related to the plasma concentration of the prodrug (*C*), the third one corresponds to the plasma concentration of the active metabolite (*C*_*m*_), and the fourth and fifth equations to *SBP* and *DBP* after treatment.


$$\frac{dX_{a}}{dt}=-K_{a}(t)*X_{a}$$ (8)

$$\frac{dC}{dt}=\frac{F}{Vd}*X_{a}*K_{a}(t)-{(Ke}_{1}(t)+K_{m}(t))*C$$ (9)

$$\frac{dC_{m}}{dt}=K_{m}(t)*C-{Ke}_{2}(t)*C_{m}$$ (10)

$$\frac{dSBP}{dt}=k_{in}(t)*\left(1-\frac{I_{max}*{C_{m}}^{n}}{{IC}_{50}^{n}+{C_{m}}^{n}}\right)-k_{out1}*SBP$$ (11)

$$\frac{dDBP}{dt}=k_{in}(t)*\left(1-\frac{I_{max}*{C_{m}}^{n}}{{IC}_{50}^{n}+{C_{m}}^{n}}\right)-k_{out2}*DBP$$ (12)

The prodrug model contains a larger number of pharmacokinetic constants than the non-prodrug model. The equation that describes the plasma concentration of the prodrug (Eq 9) depends on three constants: the absorption constant *Ka(t)*, the elimination constant of the prodrug *Ke*_*1*_*(t)*, and the metabolism constant of the prodrug *Km(t)*. The equation that describes the plasma concentration of the active metabolite (Eq 10) depends on the metabolism constant *Km(t)* and the elimination constant of the active metabolite *Ke*_*2*_*(t)*. Besides, in this case, the *SBP* and *DBP* effect equations depend on the concentration of the active metabolite *Cm*.

In both cases, non-prodrug and prodrug models, the incorporation of the circadian rhythm in the pharmacokinetic processes of each medication was performed by including circadian periodic functions for each kinetic constant *K*_*x*_*(t)* (such as *Ka(t)* and *Ke(t)*) and combinations of them (Eq 13). However, to avoid overfitting, the decision to incorporate none, only one or two kinetic constants as periodic functions was evaluated for each model.


$$K_{x}(t)=K_{x}+A_{x}\cos\left(W_{24}(t+Ta)+O_{x}\right)$$ (13)

Thus, each kinetic constant *K*_*x*_*(t)* that includes circadian rhythm has a component with a period of 24 hours (angular frequency, *W*_*24*_ = 2π/24). *K*_*x*_*(t)* depends on the integration time *t* and it is determined by the parameters *K*_*x*_ (mean pharmacokinetic constant baseline), *A*_*x*_ (amplitude of the pharmacokinetic periodic function), *O*_*x*_ *(*peak time or acrophase of the pharmacokinetic periodic function) and the dosing time (*Ta*).

### Parameter estimation and model selection

Parameter estimation for prodrug and non-prodrug models was performed through the global optimization algorithm MEIGO [77] using Eq 14 as the objective function:

$$F_{obj}=\frac{1}{k*N}\sum_{i=1}^{k}\sum_{n=1}^{N}{\left(\frac{y_{\exp\;i}(t_{n})-y_{model\;i}(t_{n})}{\sigma_{i}(t_{n})}\right)}^{2}$$ (14)

Where N is the total number of mean experimental data per variable for each *Ta*, *k* is the number of variables, and *y*_*exp i*_ is the mean experimental value of the *i* variable, *y*_*model i*_ is the value predicted by the model and *σ*_*i*_ is the standard error from the data of the *i* variable.

PK-PD models of antihypertensive medications were set by calculating the Log-Likelihood (*LogL*) and *AICc* values (Eqs 15 and 16) and selecting those with the smallest *AICc* [36].


$$LogL=-\frac{N}{2}\log(2\pi )-\sum_{i=1}^{k}\sum_{n=1}^{N}log(\sigma_{i}(t_{n}))-\frac{1}{2}\sum_{i=1}^{k}\sum_{n=1}^{N}{\left(\frac{y_{\exp\;i}(t_{n})-y_{model\;i}(t_{n})}{\sigma_{i}(t_{n})}\right)}^{2}$$ (15)

$$AICc=2n_{pars}-2LogL+2n_{pars}\left(\frac{n_{pars}+1}{N-n_{pars}-1}\right)$$ (16)

Where, *n*_*pars*_ corresponds to the number of estimated parameters of the selected model. Finally, estimation of BP parameters before treatment and BP of dipper and non-dipper patients was the same as described for the estimation of the PK-PD model parameters, using least squares as the objective function. All calculations and analyses were implemented in MATLAB 2021.

### Sensitivity and identifiability analysis

Sensitivity and identifiability analyses were performed for each model using the SENS_SYS third-party MATLAB function, which allows calculating the local sensitivity trajectory (*S*_*ij*_(*t*_*n*_)) for each variable *i* and parameter *j* at the time point (*t*_*n*_) described in Eq 17 [35]. According to the methodology described by Cortés-Ríos and Rodriguez-Fernandez [78], normalized local sensitivity trajectories (*Srel*_*ij*_(*t*_*n*_)) of each predicted variable *y*_*model i*_ with respect to each parameter *p*_*j*_ for the best parameter sets at the time point (*t*_*n*_) were obtained using the Eqs 17 and 18.


$$S_{ij}(t_{n})=\frac{dy_{model\;i}(t_{n})}{dp_{j}}$$ (17)

$${Srel}_{ij}(t_{n})=\frac{dy_{model\;i}(t_{n})}{dp_{j}}*\frac{p_{j}}{y_{model\;i}(t_{n})}$$ (18)

In order to summarize the large number of sensitivity values (for each time and variable), *δ*_*j*_ was calculated for each parameter using Eq 19 [79].


$$\delta_{j}=\sqrt{\frac{1}{k*N}\sum_{i=1}^{k}\sum_{n=1}^{N}{Srel}_{ij}^{2}(t_{n})}$$ (19)

Then, local identifiability analysis was carried out using the Fisher information matrix (*FIM*), obtained from the local sensitivity matrices *S*(*t*_*n*_) (Eq 17) and the covariance matrix (*Q*) (see Eq 20) [80].

$$FIM=\sum_{n=1}^{N}S(t_{n})\cdot Q(t_{n})\cdot S(t_{n}{)}^{T}$$ (20)

where *Q*(*t*_*n*_) is a square diagonal matrix calculated using the standard error of the data for each variable at each time point (*t*_*n*_). Since, the inverse of the FIM represents an approximation of the parameter estimation error covariance between the parameters j and h ($\sigma_{jh}^{2}={\left(FIM^{-1}\right)}_{jh}$), the diagonal of the inverse of the FIM $\left(\sigma_{jj}^{2}\right)$ is an approximation of the variance of the parameters [4,80]. Thus, assuming a normal distribution, the 95% confidence intervals (*CI*) of a parameter can be approximated by *p*±1.96 *σ*_*jj*_ [81].


$$\kappa_{jh}=\frac{\sigma_{jh}^{2}}{\sigma_{hh}\cdot \sigma_{jj}}$$ (21)

Finally, the correlation between parameters j and h (*κ*_*jh*_) can be calculated using the Eq 21 described by Ljung et al. [80].

### Dosing time optimization

Once the model parameters for each medication were estimated, simulations of BP profiles after treatment for dipper and non-dipper patterns were obtained for dosing times (*Ta*) between 0–24 hours. Data for dipper and non-dipper patterns were obtained from Hermida et al. [32] and were used to estimate the parameters of Eqs 1 and 2. These parameters were used in the previously calibrated models for each antihypertensive medication—Eqs 6 and 7 in the case of non-prodrug models and Eqs 7 and 8 for prodrug models. Then, BP profiles allowed calculating dipper percentages (Eq 22), reduced BP averages in mmHg (*BP*_*reduced*_) (Eq 23) and average peaks reduction in mmHg (*BP*_*peaks*_) (Eq 24) at different dosing times for *SBP* and *DBP*.


$$Dipper\;\left(\%\right)=\left(\frac{{BP}_{awake}-{BP}_{sleep}}{{BP}_{awake}}\right)*100$$ (22)

$${BP}_{reduced}=\frac{1}{N}\sum_{n=1}^{N}\left({BP}_{before}(t_{n})-{BP}_{after}(t_{n})\right)$$ (23)

$${BP}_{peaks}=\frac{1}{P}\sum_{p=1}^{P}\left({BP}_{before}(t_{p})-{BP}_{after}(t_{p})\right)$$ (24)

*BP*_*awake*_ is the average BP during the day (mmHg), *BP*_sleep_ is the average BP during the night rest (mmHg), *BP*_*before*(*t*)_ is the average BP value before treatment at time *t*, *BP*_*after*(*t*)_ is the average BP value after treatment at time *t*, *N* is the number of experimental data, and *P* is the number of experimental data that exceed the average BP value before treatment (*BP*_*before*_(*t*)>*mean*(*BP*_*before*_)).

Simulations allowed finding the dosing times that reach the therapeutic target BP <130/80 mmHg. Therefore, optimal dosing times were established by comparing the time in which dipper percentages were between 10–20% and the BP remained <130/80 mmHg (therapeutic objective set by ACC / AHA and WHO guidelines [8,82]). When optimal dipper percentage is not achieved (dipper percentage <10 or >20%), the dosing time at which the maximum/minimum dipper percentage was obtained is reported. Moreover, when *SBP* or *DBP* optimal values are not obtained, the *Ta* at which the minimum value of S*BP*_*max*_ or *DBP*_*max*_ is obtained (i.e., the lowest BP of the maximum BP values of the profiles) is reported. In the same way, optimal dosing time results for a different target BP can be obtained by modifying the *Target_BP* variable of the *OptimBP* function, available in the developed MATLAB codes (https://www.synapse.org/#!Synapse:syn36744682/files/).

## Supporting information

S1 Text Supplementary Figures and Tables. (PDF)
