# Supplementary material for: Dosing time optimization of antihypertensive medications by including the circadian rhythm in pharmacokinetic-pharmacodynamic models
Source: PLoS Comput Biol. 2022 Nov 14;18(11):e1010711. doi: 10.1371/journal.pcbi.1010711 (PMC9704759; doi:10.1371/journal.pcbi.1010711)
Supplement: S1 Text — (PDF) [file pcbi.1010711.s001.pdf]

Supporting Information for

Dosing time optimization of antihypertensive medications by including the circadian rhythm in pharmacokinetic-pharmacodynamic models

Javiera Cortés-Ríos[1], Ramón C. Hermida[2,3] and Maria Rodriguez-Fernandez[1*]

[1]Institute for Biological and Medical Engineering, Schools of Engineering, Medicine and Biological Sciences, Pontificia Universidad Católica de Chile, Chile.

[2]Bioengineering & Chronobiology Laboratories, Atlantic Research Center for Telecommunication Technologies (atlanTTic), Universidade de Vigo, Vigo, Spain.

[3]Bioengineering & Chronobiology Research Group. Galicia Sur Health Research Institute (IIS Galicia Sur). SERGAS-UVIGO, Vigo, Spain.

* marodriguezf@uc.cl

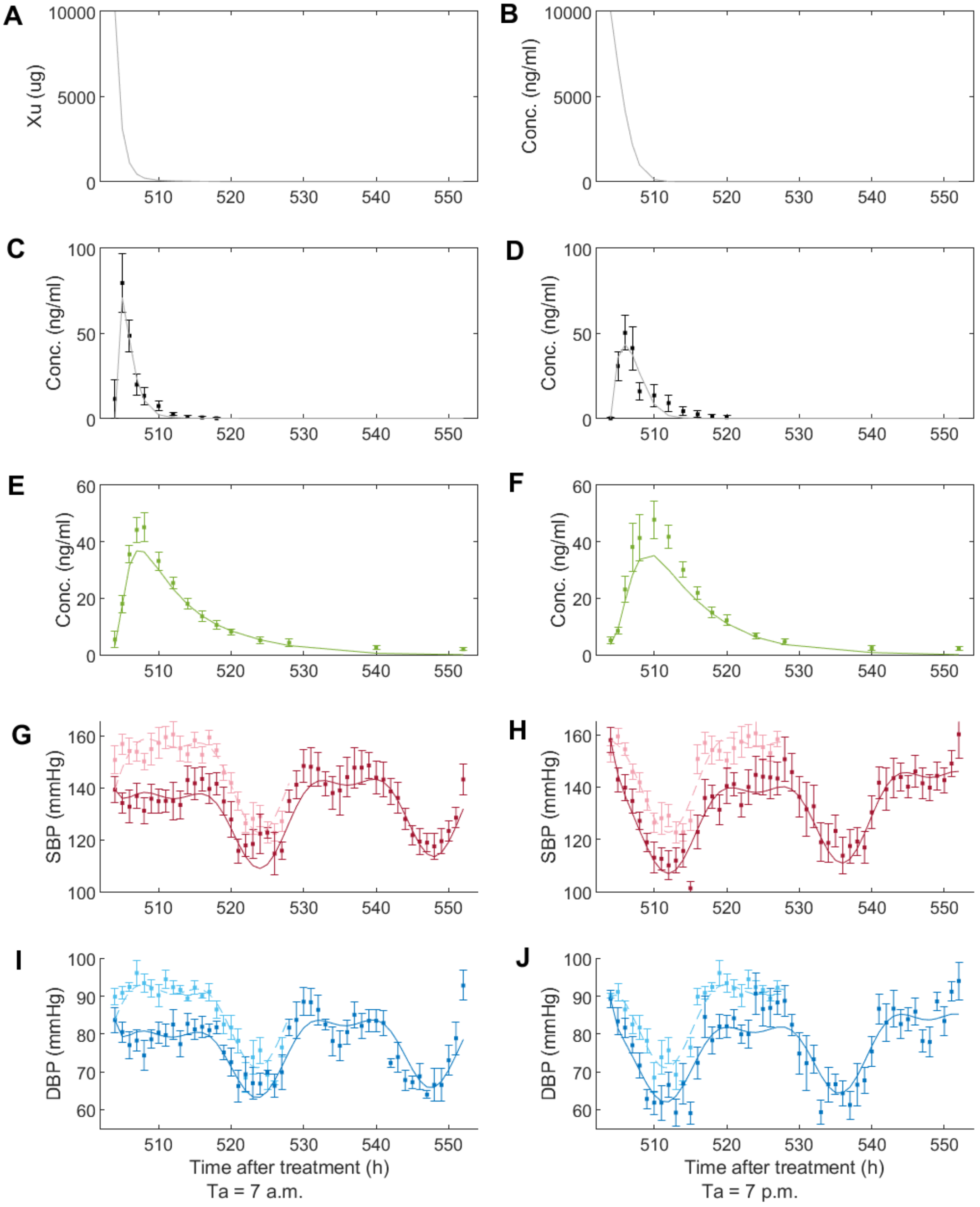

Figure A. Simulations and data for enalapril model. Panels on the left correspond to 7 a.m. dosing time and panels on the right to 7 p.m. dosing time. Figures A-B, amount available to be absorbed (Xu); C-D, enalapril concentration (data in black dots); E-F, enalaprilat concentration (data in green dots); G-H, SBP (data before treatment in light red dots and after treatment in dark red dots); I-J, DBP (data before treatment in light blue dots and after treatment in dark blue dots), in all figures the solid line represents the prediction of the model after treatment and the dotted line the prediction of the model before treatment. Data from Witte et al. [1].

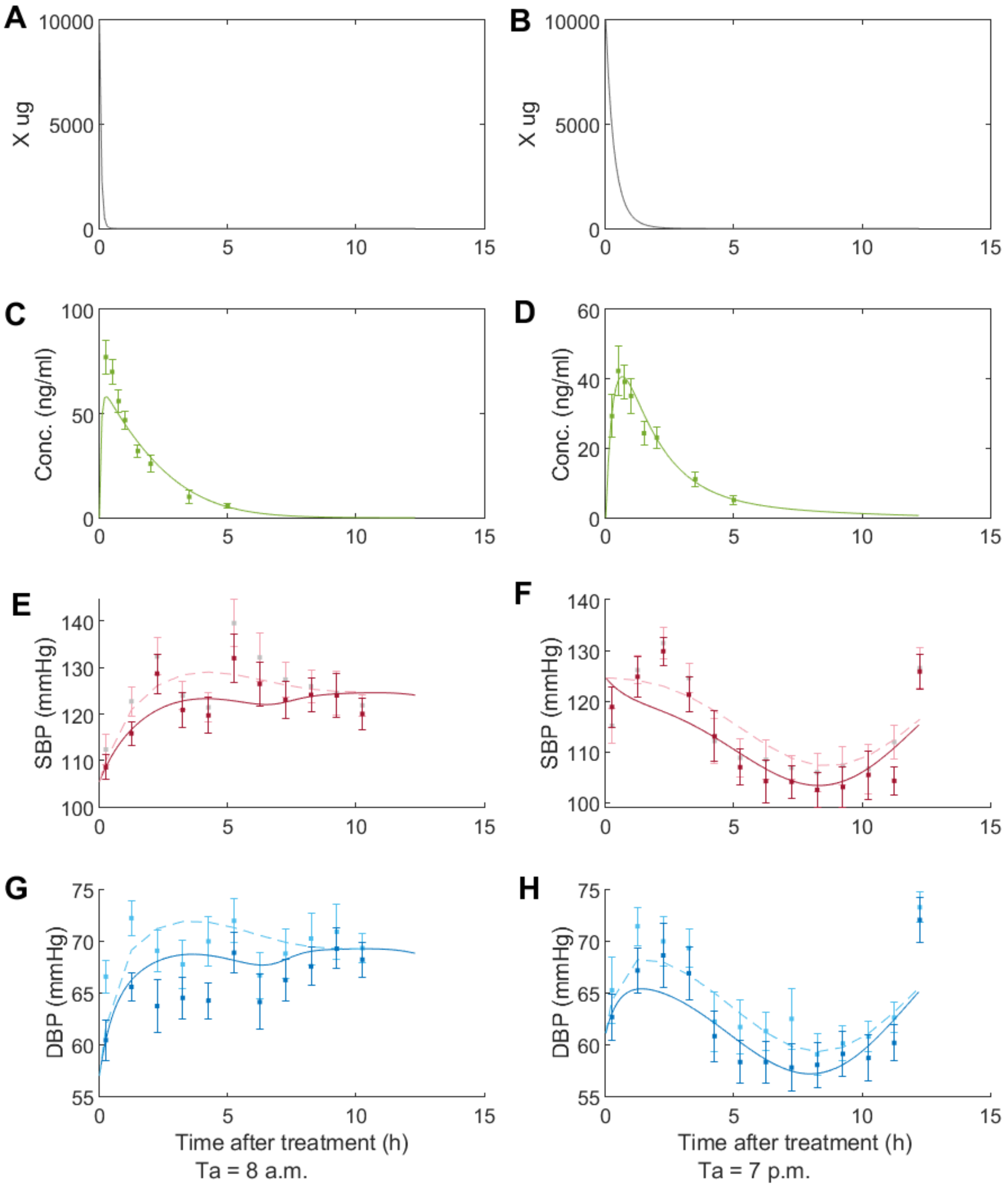

Figure B. Simulations and data for nifedipine model. Panels on the left correspond to 8 a.m. dosing time and panels on the right to 7 p.m. dosing time. Figures A-B, amount available to be absorbed (Xu); C-D, nifedipine concentration (data in green dots); E-F, SBP (data before treatment in light red dots and after treatment in dark red dots); G-H, DBP (data before treatment in light blue dots and after treatment in dark blue dots), in all figures the solid line represents the prediction of the model after treatment and the dotted line the prediction of the model before treatment. Data from Lemmer et al. [2].

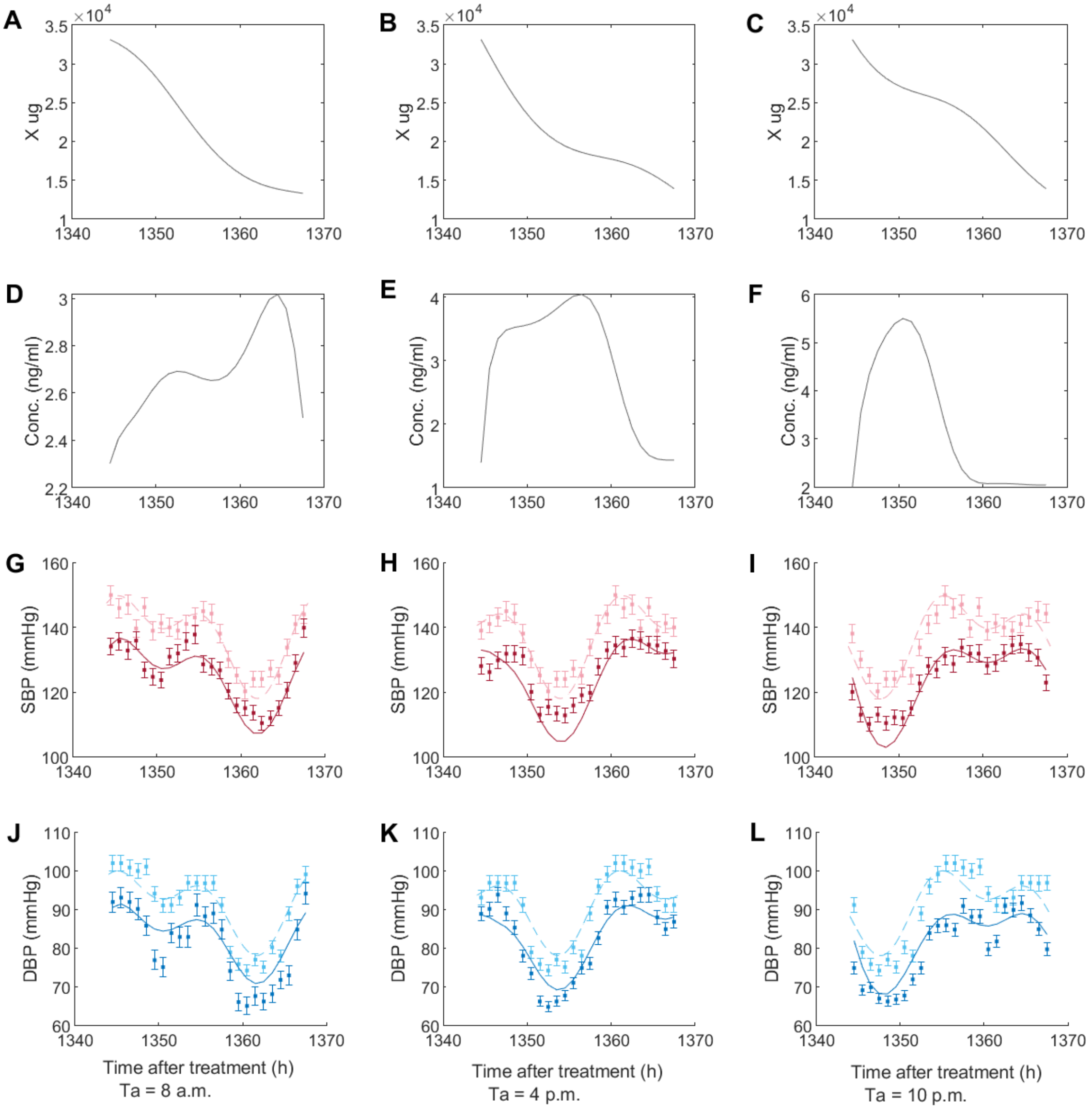

Figure C. Simulations and data for lisinopril model. Panels on the left correspond to 8 a.m. dosing time, panels in the middle to 4 p.m. dosing time and panels on the right to 10 p.m. dosing time. Figures A-B, amount available to be absorbed (Xu); C-D, lisinopril concentration (data in black dots); E-F, SBP; G-H, DBP (data before treatment in gray dots and after treatment in blue dots), in all figures the solid gray line represents the prediction of the model after treatment and the gray dotted line the prediction of the model before treatment. Data from Macchiarulo et al. [3].

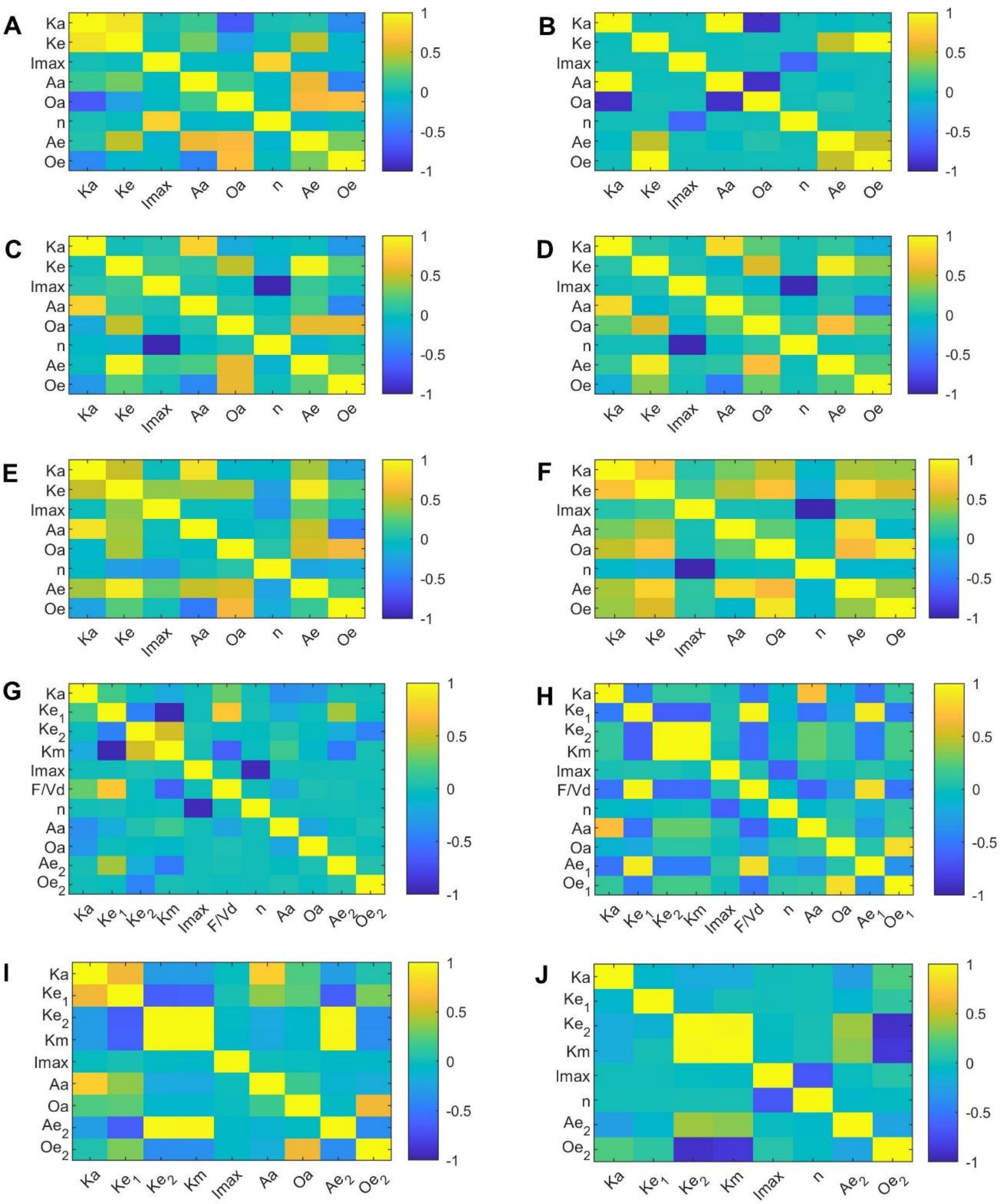

Figure D. Parameter correlation matrices of all models. Figure A, amlodipine; B, nifedipine; C, telmisartan; D, olmesartan; E, valsartan; F, lisinopril; G, enalapril; H, ramipril; I, spirapril; J, perindopril.

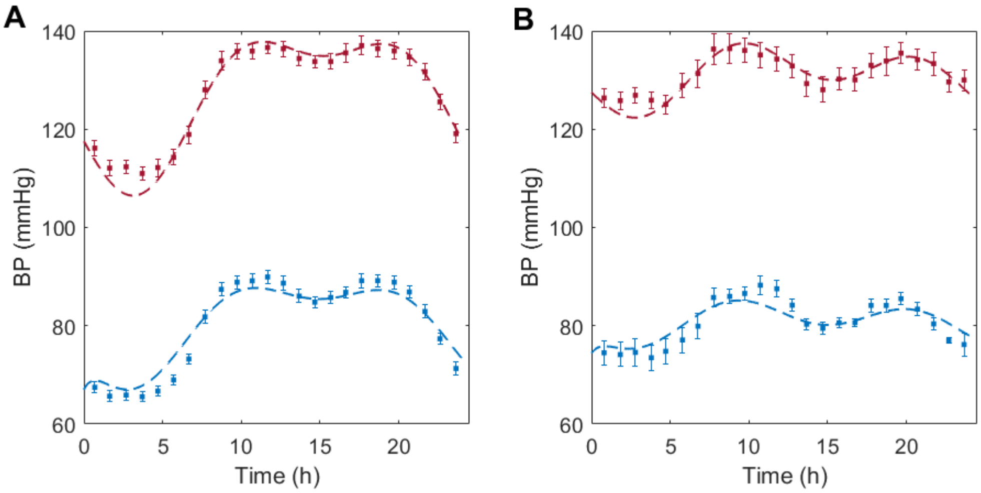

Figure E. Model simulation and BP data of dipper and non-dipper subjects. Figure A, dipper subjects; Figure B, non-dipper subjects. SBP and DBP is showed in red and blue dots, respectively. In both figures the dotted line represents the prediction of the model before treatment. Data from Hermida et al. [4].

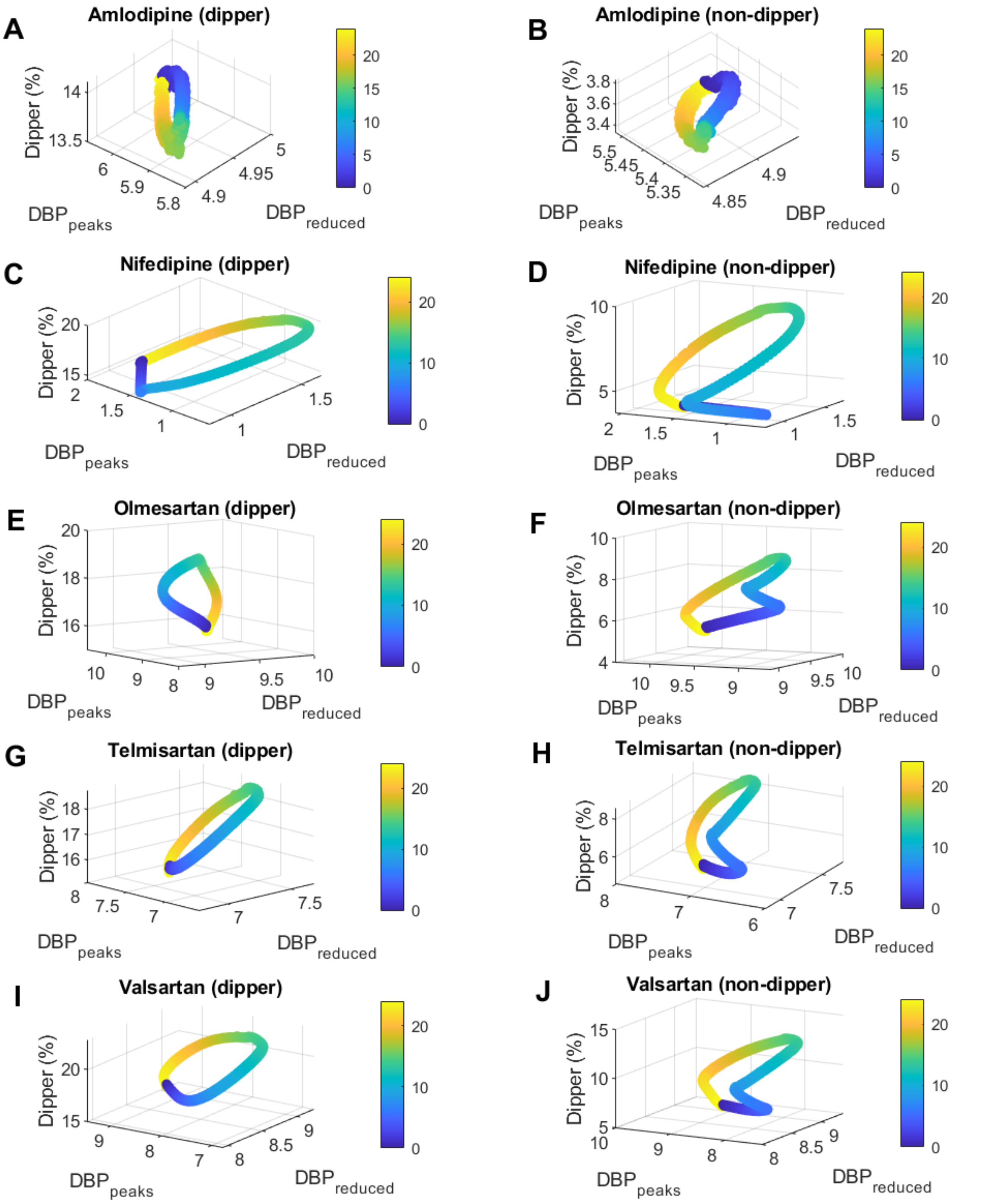

Figure F. Relationship between dosing time and DBP dipper percentage, average DBP reduction ($DBP_{reduced}$), and average DBP peaks reduction (DBP_{peaks}) for amlodipine, nifedipine, telmisartan, olmesartan, and valsartan. Panels on the left show the results for dipper subjects and those on the right for non-dipper subjects. Figure A-B, amlodipine; C-D, nifedipine; E-F, olmesartan; G-H, telmisartan; I-J, valsartan. Dosing time is represented in colors from 0 to 24 hours after awakening (colorbar).

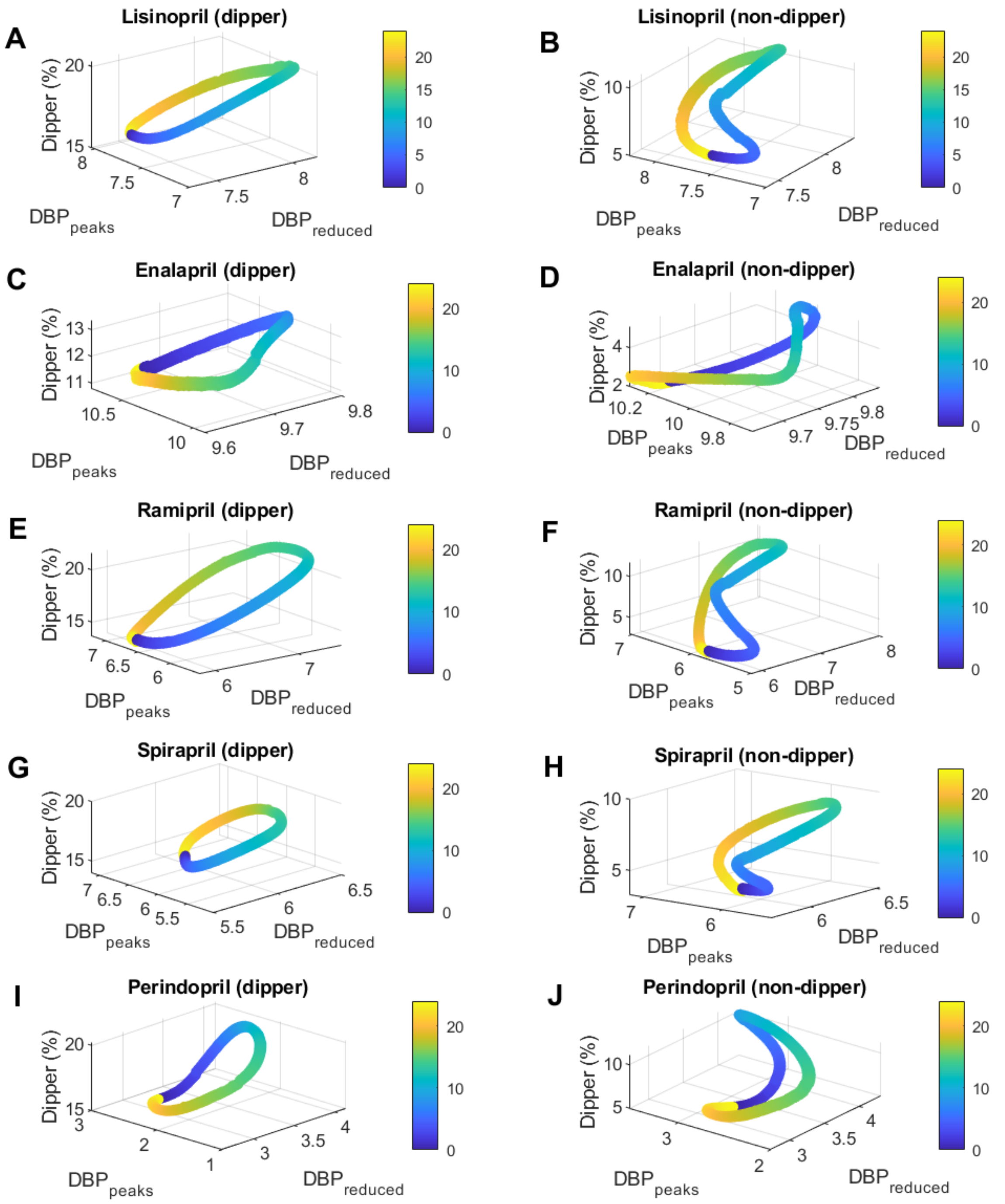

Figure G. Relationship between dosing time and DBP dipper percentage, average DBP reduction ($DBP_{reduced}$), and average DBP peaks reduction (DBP_{peaks}) for lisinopril, enalapril, ramipril, spirapril and perindopril. Panels on the left show the results for dipper subjects and those on the right for non-dipper subjects. Figure A-B, lisinopril; C-D, enalapril; E-F, ramipril; G-H, spirapril; I-J, perindopril. Dosing time is represented in colors from 0 to 24 hours after awakening (colorbar).

Table A. Estimated parameters for systolic and diastolic blood pressure before treatment for amlodipine, nifedipine, valsartan, olmesartan, and telmisartan (AM/PM experimental groups).

	Amlodipine		Nifedipine		Valsartan				Olmesartan				Telmisartan			
	AM/PM	CI *	AM/PM	CI *	AM	CI *	PM	CI *	AM	CI *	PM	CI *	AM	CI *	PM	CI *
M	153.012	440.7	133.737	309.46	141.387	133.02	134.212	142.37	121.029	80.957	125.628	110.22	123.412	66.353	122.064	87.19
A_{12}	5.772	14.199	4.679	9.996	5.502	4.599	4.048	3.743	7.18	4.078	5.081	3.916	6.197	2.84	5.566	3.344
O_{12}	-54.058	1.097	-42.555	0.661	-29.848	0.326	-23.486	0.396	-36.377	0.252	-36.161	0.312	-48.748	0.21	-48.793	0.272
A_{24}	12.816	35.282	10.794	24.925	5.416	5.277	3.245	3.509	11.542	7.488	8.992	7.754	11.096	5.756	9.346	6.451
O_{24}	-53.854	0.611	-53.95	0.413	-98.113	0.22	-98.199	0.3	-123.3	0.148	-110.77	0.19	-47.845	0.124	-47.935	0.168
$kout_1$	1.095	3.154	1.112	2.58	1.024	0.965	0.979	1.039	0.942	0.631	0.968	0.85	0.937	0.504	0.922	0.659
$kout_2$	1.689	4.864	2.002	4.632	1.664	1.568	1.58	1.678	1.48	0.99	1.531	1.345	1.463	0.787	1.475	1.055

* Confidence Interval (CI) for each parameter, the CI units are the same as the corresponding parameter value.

Table B. Estimated parameters for systolic and diastolic blood pressure before treatment for lisinopril, ramipril, enalapril, spirapril and perindopril (AM/PM experimental groups).

	Lisinopril		Ramipril				Enalapril		Spirapril				Perindopril	
	AM/PM	CI *	AM	CI *	PM	CI *	AM/PM	CI *	AM	CI *	PM	CI *	AM/PM	CI *
M	144.576	156.09	141.014	114.08	142.991	134.02	146.369	241.76	142.636	68.34	135.04	123.62	144.727	221.51
A_{12}	9.413	9.02	6.929	5.126	5.774	4.654	7.758	11.155	7.36	3.149	5.611	4.464	4.724	6.623
O_{12}	-98.079	0.391	-48.718	0.266	-48.901	0.347	-54.53	0.619	-61.296	0.143	-55.1	0.325	-35.449	0.567
A_{24}	12.222	12.928	14.432	11.585	12.636	11.358	17.037	26.594	11.297	5.5	9.741	8.693	8.923	13.571
O_{24}	-28.338	0.237	-54.184	0.148	-54.204	0.192	-53.894	0.322	-10.205	0.091	-16.579	0.188	-53.485	0.345
$kout_1$	1.055	1.139	1.106	0.897	1.106	1.037	1.001	1.653	1.12	0.538	1.046	0.958	0.973	1.491
$kout_2$	1.587	1.716	1.685	1.364	1.687	1.58	1.716	2.828	1.735	0.834	1.654	1.515	1.592	2.439

* Confidence Interval (CI) for each parameter, the CI units are the same as the corresponding parameter value.

Table C. Fixed parameters for PK-PD models.

	Amlodipine	Nifedipine	Valsarta n	Olmesartan	Telmisartan	Lisinopril	Ramipril	Enalapril	Spirapril	Perindopril
IC_{50} (nM)	0.777 [5]	1.42 [5]	0.24 [6]	0.058 [6]	0.17 [6]	7.46 [7]	0.295*[8]	0.676*[9]	0.373*[10]	0.866*[11]
F (%)	64 [12]	89 [13]	23 [14]	28.6 [15]	43 [14]	25 [16]	50-60**[17]	60 [17]	50 [16]	95 [18]
Vd (L)	1425.2 [12]	139.43 [19]	17 [20]	34.9 [21]	500 [14]	124 [22]	-	-	28 [16]	6.4 [23]
F/Vd	0.000449	0.00638	0.0132	0.00819	0.00086	0.00202	-	-	0.0179	0.1484

* IC_{50} value of active metabolites (ramiprilat, enalaprilat, spiraprilat, perindoprilat).
** The average value (55%) was used.

Table D. Estimated parameters for non-prodrug selected models.

	Amlodipino		Nifedipino		Valsartan		Olmesartan		Telmisartan		Lisinopril	
	value	CI *	value	CI *	value	CI *	value	CI *	value	CI *	value	CI *
Ka	0.01	0.003	8.667	0.969	0.048	0.004	0.084	0.003	0.086	0.004	0.039	0.002
Ke	0.415	0.098	0.498	0.7	408.161	25.745	24.088	1.455	6.729	0.359	0.637	0.041
Aa	0.01	0.003	6.399	1.018	0.04	0.004	0.073	0.004	0.078	0.006	-48.919	0.238
Oa	-45.571	0.399	-40.051	0.052	-87.101	0.085	-100.104	0.057	-105.507	0.068	0.986	0.263
Ae	0.39	0.113	0.254	0.129	358.152	30.059	19.421	1.737	6.399	0.434	0.475	0.09
Oe	-6.914	0.254	-10.582	3.072	-4.882	0.079	-105.448	0.076	-10.67	0.048	-16.977	0.171
Imax	0.178	0.027	0.044	0.023	0.229	0.008	0.169	0.023	0.14	0.019	0.345	0.078
n	0.683	0.248	3.314	29.826	1.222	0.154	0.617	0.386	0.709	0.46	0.026	0.005

* Confidence Interval (CI) for each parameter, the CI units are the same as the corresponding parameter value.

Table E. Estimated parameters for prodrug selected models.

	Ramipril		Enalapril		Spirapril		Perindopril	
	value	CI *	value	CI *	value	CI *	value	CI *
Ka	0.079	0.004	0.77	0.001	0.046	0.003	0.1	0.003
Ke_1	1.06	0.252	0.74	0.023	3	0.212	1.284	0.052
Ke_2	3.364	2.626	0.127	0.002	1.96	1.498	0.372	0.103
Km	0.032	0.025	0.288	0.019	0.125	0.096	0.013	0.004
Aa	0.079	0.008	0.595	0.008	0.024	0.003	-	-
Oa	-67.451	0.098	-107.938	0.01	-30.633	0.095	-	-
Ae_1	0.984	0.199	-	-	-	-	-	-
Oe_1	-85.747	0.143	-	-	-	-	-	-
Ae_2	-	-	0.013	0.009	0.946	0.812	0.356	0.031
Oe_2	-	-	-60.056	0.701	-30.193	0.146	-9.363	0.164
F/Vd **	0.171	0.041	0.018	0.001	-	-	-	-
Imax	0.166	0.009	0.171	0.022	0.387	0.021	0.103	0.016
n	1.505	0.338	0.293	0.148	-	-	2.699	1.751

* Confidence Interval (CI) for each parameter, the CI units are the same as the corresponding parameter value.
** Estimated parameter for enalapril neither ramipril since there are no reported values of volume of distribution (Vd).

Table F. Estimated parameters for systolic and diastolic blood pressure before treatment for dipper and non-dipper patterns[ϕ].

	Dipper		Non-dipper	
	value	CI *	value	CI *
M	129.55	82.221	144.92	262.563
A_{12}	7.33	3.97	5.73	8.831
O_{12}	-35.73	0.246	-35.67	0.619
A_{24}	14.93	9.038	4.64	7.919
O_{24}	-53.96	0.14	-53.61	0.401
$kout_1$	1.02	0.646	1.11	2.003
$kout_2$	1.61	1.02	1.79	3.246

ϕ Parameters were adjusted to equations 1 and 2 using blood pressure data of dipper and non-dipper hypertensive subjects from the work of Hermida et al.[4].

* Confidence Interval (CI) for each parameter, the CI units are the same as the corresponding parameter value.

Table G. Dosing times after awakening for maximum (Ta_{max}) and minimum (Ta_{min}) values of dipper percentage (Dip), SBP average reduction ($SBP_{reduced}$), and SBP average peaks reduction (SBP_{peaks}) for dipper subjects.

Drug/ prodrug	Dipper Percentage					Average Reduction					Average Peaks Reduction (mmHg)				
	Dip_{min} (%)	Ta_{min} (hh:mm)	Dip_{max} (%)	Ta_{max} (hh:mm)	ΔDip (%)	$SBP_{reduced,min}$ (mmHg)	Ta_{min} (hh:mm)	$SBP_{reduced,max}$ (mmHg)	Ta_{max} (hh:mm)	$\Delta SBP_{reduced}$ (mmHg)	$SBP_{peaks,min}$ (mmHg)	Ta_{min} (hh:mm)	$SBP_{peaks,max}$ (mmHg)	Ta_{max} (hh:mm)	ΔSBP_{peaks} (mmHg)
Amlodipino	12.63	00:01	13.22	13:24	0.59	7.71	16:36	7.87	01:21	0.16	9.15	14:27	9.54	00:47	0.39
Nifedipino	14.2	23:55	18.92	13:30	4.72	1.34	06:22	2.59	15:31	1.25	0.92	12:58	2.98	23:59	2.06
Lisinopril	15.27	21:49	19.44	13:06	4.17	11.54	04:04	12.87	15:29	1.33	11.13	11:47	12.65	19:29	1.52
Enalapril	10.09	22:25	12.54	09:24	2.45	15.18	16:45	15.45	06:16	0.27	15.64	13:27	16.85	23:12	1.21
Ramipril	13.75	23:06	21.32	12:25	7.57	9.16	03:14	11.87	14:21	2.71	8.72	09:05	11.39	16:59	2.67
Perindopril	15.22	00:01	19.78	11:54	4.56	4.11	19:11	6.53	07:34	2.42	1.99	14:47	4.4	05:52	2.41
Spirapril	13.98	22:48	19.38	13:31	5.4	8.94	06:19	10.2	18:42	1.26	8	12:16	11.2	21:41	3.2
Telmisartan	15.06	23:36	18.51	13:01	3.45	10.69	05:26	12.11	17:38	1.42	10.36	08:11	12.59	20:29	2.23
Olmesartan	15.37	23:42	19	13:07	3.63	13.81	09:06	15.61	21:45	1.8	13.06	10:52	16.52	22:47	3.46
Valsartan	16.05	23:42	22.3	13:49	6.25	12.49	07:03	14.59	19:35	2.1	10.67	11:47	14.86	22:29	4.19

Table H. Dosing times after awakening for maximum (Ta_{max}) and minimum (Ta_{min}) values of dipper percentage (Dip), DBP average reduction ($DBP_{reduced}$), and DBP average peaks reduction (DBP_{peaks}) for dipper subjects.

Drug/ prodrug	Dipper Percentage					Average Reduction (mmHg)					Average Peaks Reduction (mmHg)				
	Dip_{min} (%)	Ta_{min} (hh:mm)	Dip_{max} (%)	Ta_{max} (hh:mm)	ΔDip (%)	$DBP_{reduced,min}$ (mmHg)	Ta_{min} (hh:mm)	$DBP_{reduced,max}$ (mmHg)	Ta_{max} (hh:mm)	$\Delta DBP_{reduced}$ (mmHg)	$DBP_{peaks,min}$ (mmHg)	Ta_{min} (hh:mm)	$DBP_{peaks,max}$ (mmHg)	Ta_{max} (hh:mm)	ΔDBP_{peaks} (mmHg)
Amlodipino	13.54	00:01	14.11	13:24	0.57	4.89	16:32	4.99	01:21	0.1	5.81	15:11	6.07	00:47	0.26
Nifedipino	14.59	00:00	19.35	13:43	4.76	0.85	06:22	1.64	15:31	0.79	0.57	12:53	1.92	23:59	1.35
Lisinopril	15.96	22:19	20.26	13:18	4.3	7.31	04:04	8.16	15:29	0.85	7.06	12:06	8.03	19:31	0.97
Enalapril	10.8	22:43	13.34	09:54	2.54	9.63	16:45	9.8	06:15	0.17	9.92	13:27	10.72	23:12	0.8
Ramipril	13.66	23:12	21.55	12:49	7.89	5.81	03:04	7.53	14:21	1.72	5.54	08:59	7.22	16:54	1.68
Perindopril	15.48	00:36	20.17	12:13	4.69	2.61	19:11	4.14	07:34	1.53	1.24	14:36	2.78	05:52	1.54
Spirapril	13.99	23:00	19.68	13:49	5.69	5.67	06:19	6.47	18:42	0.8	5.06	12:29	7.14	21:30	2.08
Telmisartan	15.11	23:42	18.73	13:43	3.62	6.78	05:26	7.68	17:51	0.9	6.58	08:11	7.99	20:54	1.41
Olmesartan	15.42	23:48	19.21	13:37	3.79	8.76	09:06	9.9	21:45	1.14	8.31	11:00	10.51	22:43	2.2
Valsartan	16.2	23:48	22.79	14:13	6.59	7.92	07:03	9.25	19:35	1.33	6.77	11:54	9.47	22:18	2.7

Table I. Dosing times after awakening for maximum (Ta_{max}) and minimum (Ta_{min}) values of dipper percentage (Dip), SBP average reduction ($SBP_{reduced}$), and SBP average peaks reduction (SBP_{peaks}) for non-dipper subjects.

Drug/ prodrug	Dipper Percentage					Average Reduction (mmHg)					Average Peaks Reduction (mmHg)				
	Dip_{min} (%)	Ta_{min} (hh:mm)	Dip_{max} (%)	Ta_{max} (hh:mm)	ΔDip (%)	$SBP_{reduced,min}$ (mmHg)	Ta_{min} (hh:mm)	$SBP_{reduced,max}$ (mmHg)	Ta_{max} (hh:mm)	$\Delta SBP_{reduced}$ (mmHg)	$SBP_{peaks,min}$ (mmHg)	Ta_{min} (hh:mm)	$SBP_{peaks,max}$ (mmHg)	Ta_{max} (hh:mm)	ΔSBP_{peaks} (mmHg)
Amlodipino	3.08	23:07	3.55	14:24	0.47	7.85	16:25	8	01:49	0.15	8.6	15:00	8.94	23:49	0.34
Nifedipino	3.68	23:54	8.93	13:31	5.25	1.29	06:31	2.8	15:17	1.51	1.17	05:11	3.24	21:56	2.07
Lisinopril	5.46	21:37	10.15	13:12	4.69	11.93	03:43	13.46	15:09	1.53	11.53	05:06	13.27	18:11	1.74
Enalapril	2.37	22:19	4.94	09:30	2.57	15.63	21:26	15.92	06:15	0.29	15.7	14:02	16.61	21:56	0.91
Ramipril	3.25	22:54	11.72	12:37	8.47	9.41	03:05	12.45	14:09	3.04	8.23	04:11	11.41	16:11	3.18
Perindopril	5.87	00:00	10.98	12:01	5.11	4.41	19:22	6.97	07:54	2.56	3.26	15:11	5.61	08:18	2.35
Spirapril	3.46	22:42	9.58	13:37	6.12	9.25	05:43	10.56	18:00	1.31	8.73	12:43	11.51	20:20	2.78
Telmisartan	4.73	23:30	8.58	13:25	3.85	11.06	05:18	12.54	17:17	1.48	10.28	05:11	12.68	19:35	2.4
Olmesartan	5.05	23:36	9.1	13:25	4.05	14.35	08:45	16.1	21:39	1.75	13.92	11:33	16.73	21:46	2.81
Valsartan	5.84	23:42	12.89	14:01	7.05	13.02	06:35	15.15	19:16	2.13	11.86	05:28	15.66	21:23	3.8

Table J. Dosing times after awakening for maximum (Ta_{max}) and minimum (Ta_{min}) values of dipper percentage (Dip), DBP average reduction ($DBP_{reduced}$), and DBP average peaks reduction (DBP_{peaks}) for non-dipper subjects.

**Drug/ prodrug**	**Dipper Percentage**					**Average Reduction (mmHg)**					**Average Peaks Reduction (mmHg)**				
	Dip_{min} (%)	Ta_{min} (hh:mm)	Dip_{max} (%)	Ta_{max} (hh:mm)	ΔDip (%)	$DBP_{reduced,min}$ (mmHg)	Ta_{min} (hh:mm)	$DBP_{reduced,max}$ (mmHg)	Ta_{max} (hh:mm)	$\Delta DBP_{reduced}$ (mmHg)	$DBP_{peaks,min}$ (mmHg)	Ta_{min} (hh:mm)	$DBP_{peaks,max}$ (mmHg)	Ta_{max} (hh:mm)	ΔDBP_{peaks} (mmHg)
Amlodipino	3.34	23:37	3.84	14:24	0.5	4.85	16:25	4.94	01:49	0.09	5.31	15:26	5.52	23:40	0.21
Nifedipino	3.7	23:54	9	13:49	5.3	0.8	06:31	1.73	15:17	0.93	0.66	05:12	2.05	21:52	1.39
Lisinopril	5.69	22:13	10.55	13:30	4.86	7.37	03:43	8.31	15:09	0.94	7.11	05:04	8.24	18:16	1.13
Enalapril	2.43	22:37	5.11	10:00	2.68	9.66	21:15	9.84	06:15	0.18	9.7	14:02	10.29	21:46	0.59
Ramipril	2.94	23:00	11.73	13:01	8.79	5.81	03:05	7.69	14:09	1.88	4.99	04:10	7.04	16:10	2.05
Perindopril	5.84	00:30	11.11	12:19	5.27	2.72	19:22	4.3	07:54	1.58	2.01	15:04	3.53	08:13	1.52
Spirapril	3.27	23:00	9.67	13:55	6.4	5.71	05:31	6.52	18:00	0.81	5.39	12:51	7.18	20:22	1.79
Telmisartan	4.57	23:36	8.59	13:55	4.02	6.83	05:27	7.75	17:17	0.92	6.31	05:04	7.85	19:34	1.54
Olmesartan	4.9	23:42	9.11	13:55	4.21	8.87	08:45	9.94	21:39	1.07	8.64	12:04	10.38	21:40	1.74
Valsartan	5.8	23:48	13.2	14:19	7.4	8.04	06:34	9.36	19:16	1.32	7.3	05:10	9.76	21:16	2.46

SI References

1. Witte K, Weisser K, Neubeck M, Mutschler E, Lehmann K, Hopf R, et al. Cardiovascular effects, pharmacokinetics, and converting enzyme inhibition of enalapril after morning versus evening administration. Clin Pharmacol Ther. 1993;54: 177–186. doi:10.1038/clpt.1993.129
2. Lemmer B, Nold G, Behne S, Kaiser R. Chronopharmacokinetics and cardiovascular effects of nifedipine. Chronobiol Int. 1991;8: 485–494. doi:10.3109/07420529109059184
3. C. Macchiarulo, R. Pieri, D. Chieppa Mitolo, A. Pirrelli. Management of antihypertensive treatment with Lisinopril: a chronotherapeutic approach - PubMed. Eur Rev Med Pharmacol Sci. 1999;3: 269–275.
4. Hermida RC, Calvo C, Ayala DE, Mojón A, López JE. Relationship between physical activity and blood pressure in dipper and non-dipper hypertensive patients. J Hypertens. 2002;20: 1097–104. doi:10.1097/00004872-200206000-00020
5. Burges RA, Gardiner DG, Gwilt M, Higgins AJ, Blackburn KJ, Campbell SF, et al. Calcium channel blocking properties of amlodipine in vascular smooth muscle and cardiac muscle in vitro: Evidence for voltage modulation of vascular dihydropyridine receptors. J Cardiovasc Pharmacol. 1987;9: 110–119. doi:10.1097/00005344-198701000-00018
6. Mire DE, Silfani TN, Pugsley MK. A review of the structural and functional features of olmesartan medoxomil, an angiotensin receptor blocker. J Cardiovasc Pharmacol. 2005;46: 585–593. doi:10.1097/01.FJC.0000180902.78230.FD
7. Shionoiri H, Minamisawa K, Ueda SI, Abe Y, Ebina T, Sugimoto KI, et al. Pharmacokinetics and antihypertensive effects of lisinopril in hypertensive patients with normal and impaired renal function. J Cardiovasc Pharmacol. 1990;16: 594–600. doi:10.1097/00005344-199010000-00010
8. Ceconi C, Francolini G, Olivares A, Comini L, Bachetti T, Ferrari R. Angiotensin-converting enzyme (ACE) inhibitors have different selectivity for bradykinin binding sites of human somatic ACE. Eur J Pharmacol. 2007;577: 1–6. doi:10.1016/J.EJPHAR.2007.07.061
9. Donnelly R, Meredith PA, Elliott HL, Reid JL. Kinetic-dynamic relations and individual responses to enalapril. Hypertension. 1990;15: 301–309. doi:10.1161/01.HYP.15.3.301
10. Smith EM, Sybertz EJ, Baum T, Swiss GF, Neustadt BR, Mcnamara P, et al. Angiotensin converting enzyme inhibitors: spirapril and related compounds. J Med Chem. 1989;32: 1600–1606. doi:10.1021/JM00127A033
11. Barthelmebs M, Devissaguet M, Imbs JL. Antihypertensive effect of perindopril: Experimental pharmacology. Clin Exp Hypertens - Part A Theory Pract. 1989;11: 471–486.
12. Faulkner J, McGibney D, Chasseaud L, Perry J, Taylor I. The pharmacokinetics of amlodipine in healthy volunteers after single intravenous and oral doses and after 14 repeated oral doses given once daily. Br J Clin Pharmacol. 1986;22: 21. doi:10.1111/J.1365-2125.1986.TB02874.X
13. Khan KM, Patel JB, Schaefer TJ. Nifedipine. xPharm Compr Pharmacol Ref. 2022; 1–6. doi:10.1016/B978-008055232-3.62280-9
14. Kirch W, Horn B, Schweizer J. Comparison of angiotensin II receptor antagonists. Eur J Clin Invest. 2001;31: 698–706. doi:10.1046/J.1365-2362.2001.00871.X
15. Brunner HR. The new oral angiotensin II antagonist olmesartan medoxomil: a concise overview. J Hum Hypertens. 2002;16 Suppl 2: S13–S16. doi:10.1038/SJ.JHH.1001391
16. Song JC, White CM. Clinical pharmacokinetics and selective pharmacodynamics of new angiotensin converting enzyme inhibitors: an update. Clin Pharmacokinet. 2002;41: 207–224. doi:10.2165/00003088-200241030-00005
17. Brown NJ, Vaughan DE. Angiotensin-converting enzyme inhibitors. Circulation. 1998;97: 1411–1420. doi:10.1161/01.CIR.97.14.1411
18. Devissaguet J, Ammoury N, Devissaguet M, Perret L. Pharmacokinetics of perindopril and its metabolites in healthy volunteers. Fundam Clin Pharmacol. 1990;4: 175–189. doi:10.1111/J.1472-8206.1990.TB00486.X
19. Walley T, Heagerty A, Woods K, Bing R, Pohl J, Barnett D. Pharmacokinetics and pharmacodynamics of nifedipine infusion in normal volunteers. Br J Clin Pharmacol.

1987;23: 693. doi:10.1111/J.1365-2125.1987.TB03103.X
20. Israili ZH. Clinical pharmacokinetics of angiotensin II (AT1) receptor blockers in hypertension. J Hum Hypertens. 2000;14 Suppl 1: S73–S86. doi:10.1038/SJ.JHH.1000991
21. Schwocho LR, Masonson HN. Pharmacokinetics of CS-866, a new angiotensin II receptor blocker, in healthy subjects. J Clin Pharmacol. 2001;41: 515–527. doi:10.1177/00912700122010393
22. Goa KL, Balfour JA, Zuanetti G. Lisinopril. A review of its pharmacology and clinical efficacy in the early management of acute myocardial infarction. Drugs. 1996;52: 564–588. doi:10.2165/00003495-199652040-00011
23. Parker E, Aarons L, Rowland M, Resplandy G. The pharmacokinetics of perindoprilat in normal volunteers and patients: influence of age and disease state. Eur J Pharm Sci. 2005;26: 104–113. doi:10.1016/J.EJPS.2005.05.006
